# Association Analysis of the Circulating Proteome With Sarcopenia‐Related Traits Reveals Potential Drug Targets for Sarcopenia

**DOI:** 10.1002/jcsm.13720

**Published:** 2025-02-13

**Authors:** Simin Wen, Siqi Xu, Xizeng Zong, Shifeng Wen, Wende Xiao, Weipeng Zheng, Han Cen, Zhaohua Zhu, Jingyu Xie, Yan Zhang, Changhai Ding, Guangfeng Ruan

**Affiliations:** ^1^ Department of Orthopedics, Guangzhou First People's Hospital Guangzhou Medical University Guangzhou China; ^2^ Clinical Research Centre, the Second Affiliated Hospital, School of Medicine South China University of Technology Guangzhou China; ^3^ Clinical Research Centre, Zhujiang Hospital Southern Medical University Guangzhou China; ^4^ Department of Population and Public Health Sciences, Keck School of Medicine University of Southern California Los Angeles California USA; ^5^ Menzies Institute for Medical Research University of Tasmania Hobart Australia

**Keywords:** circulating proteome, drug targets, Mendelian randomization, sarcopenia

## Abstract

**Background:**

Sarcopenia severely affects the physical health of the elderly. Currently, there is no specific drug available for sarcopenia. This study aims to identify pathogenic proteins and druggable targets for sarcopenia through Mendelian randomization (MR)–based analytical framework.

**Methods:**

A sequential stepwise screening method that includes two‐sample MR, Steiger filtering test and colocalization (MRSC) was applied to identify causal proteins associated with sarcopenia‐related traits. In the MR analyses, 4372 circulating proteins with valid instrumental variables (IVs) from eight proteomic genome‐wide association studies were utilized as exposures, and nine sarcopenia‐related traits were utilized as outcomes. IVs were classified into cis–protein quantitative trait loci (pQTLs) and trans‐pQTLs based on their positions. We conducted cis‐only MRSC analyses and cis + trans MRSC analyses using cis‐pQTLs and cis + trans pQTLs as IVs, respectively. Post‐MRSC analyses were conducted on the prioritized findings of MRSC, including annotation of protein‐altering variants (PAVs), assessment of overlap between pQTLs and expression quantitative trait loci (eQTLs), protein–protein interaction (PPI) analysis, pathway enrichment analysis and annotation of drug targets. Utilizing data from the UK Biobank, we performed an observational study to explore the associations between baseline circulating protein levels and the longitudinal changes in nine sarcopenia‐related traits.

**Results:**

A total of 181 causal associations for 65 proteins were prioritized by the cis‐only MRSC analyses and 227 associations for 91 proteins were prioritized by the cis + trans MRSC analyses. Among the prioritized proteins, the majority of them employed non‐PAVs as IVs and most of their cis‐pQTLs overlapped with corresponding eQTLs and exhibited consistent directionality, with only one trans‐pQTL overlapping with an eQTL. The PPI network of cis‐only MRSC‐prioritized proteins (*p* = 4.04 × 10^−4^) and cis + trans MRSC‐prioritized proteins (*p* = 8.76 × 10^−5^) showed significantly more interactions than expected. Reactome, KEGG and GO pathway enrichment analyses for cis‐only MRSC‐prioritized proteins identified 52, 12 and 79 enriched pathways, respectively (adjusted *p* < 0.05). For proteins identified by cis + trans MRSC analyses, only 15 pathways were enriched through the GO pathway enrichment analyses. In the observational study, 197 circulating proteins were identified to be associated with one or more sarcopenia‐related traits (*p* < 0.05/2923). Among them, the significant associations of CTSB (negative association) and ASGR1 (positive association) with sarcopenia‐related traits were observed to have consistent directional associations in both MR‐based studies and observational studies. Drug target annotations suggested that 52 MRSC‐prioritized proteins and 145 biomarkers are drug targets or druggable.

**Conclusions:**

This study identified 89 potential pathogenic proteins and 197 candidate biomarkers for sarcopenia, providing valuable clues for the development of therapeutic drugs for sarcopenia.

## 1 Introduction

Sarcopenia is a disease characterized by progressive loss of muscle mass, strength and function with aging, which increases the risk of falls, fractures and frailty in the elderly, potentially leading to severe clinical consequences and increasing the socioeconomic burden [1]. Studies have shown that the prevalence of sarcopenia in the elderly population ranges from 9.9% to 40.4%, with differences mainly due to different study populations and diagnostic criteria [2]. It is estimated that by 2050, the global population aged 60 and over will increase to 2.1 billion. Thus, even with conservative estimates of prevalence, sarcopenia will affect more than 200 million people in the future [3]. Therefore, as a global public health issue, sarcopenia has attracted widespread attention worldwide.

The aetiology of sarcopenia is attributed to the combined effects of various environmental and genetic factors. Currently, no specific drugs have been successfully developed for sarcopenia, and no definitive treatment guidelines have been established. The progression of the disease is primarily controlled through resistance training and nutritional supplementation [4]. Although it is widely believed that the major risk factor of sarcopenia is aging, nowadays, studies have suggested that the development of sarcopenia may begin at the early stage of life and is affected by many factors beyond age [5, 6, 7]. This highlights the importance and feasibility of actively preventing sarcopenia through the exploration of effective drug targets.

With the continuous development of high‐throughput sequencing technology and bioinformatics methods, the identification of potential intervention targets for diseases in bulk has become possible. Up to now, several genome‐wide association studies (GWASs) have reported potential single nucleotide polymorphisms (SNPs) associated with sarcopenia [8, 9, 10, 11]. Although these studies provide clues to uncovering the aetiology of sarcopenia and identifying drug targets, the translation of associated genetic loci into disease drug targets based solely on GWAS results requires a lengthy validation process. Proteins are the ultimate participants in various physiological and pathological processes of diseases, connecting with the functions of various organs in the body and serving as a reliable source of drug targets [12, 13, 14].

Previously, the vast majority of research on the pathogenic proteins of sarcopenia has focused only on a few specific proteins, and most studies have been unable to establish reliable causal associations due to limitations in research methods [15, 16, 17, 18, 19]. In recent years, researchers have integrated disease GWAS data with plasma proteomic GWAS data using the Mendelian randomization (MR) method to identify disease‐related protein quantitative trait loci (pQTLs) and subsequently reported potential pathogenic proteins. By directly identifying drug targets at the protein level, this method has yielded more reliable results and has been widely applied and recognized [20, 21].

MR is a widely used genetic epidemiology method that enhances the accuracy of causal inference by minimizing confounding effects and excluding potential reverse causality. The principle of MR is based on Mendel's second law, which states that genetic alleles segregate independently when DNA is transmitted from parents to offspring during gamete formation [22]. This is analogous to the random allocation in a randomized controlled trial, which aims to create groups with comparable characteristics, thereby minimizing the risk of confounding.

In this study, to precisely identify potential pathogenic proteins and druggable targets for sarcopenia on a large scale, we carried out stepwise MRSC screening analyses, followed by a series of validations, annotations and evaluations of the identified findings. Besides, we also conducted an observational study using the UK Biobank (UKB) dataset to explore the circulating biomarkers for sarcopenia. This research may provide clues for the prevention and treatment of sarcopenia, which is of significant importance in helping the elderly improve physical function and quality of life.

## 2 Methods

### 2.1 Data Sources

Exposures in the MR analyses were genetically predicted circulating proteins from eight proteomic GWASs [23, 24, 25, 26, 27, 28, 29, 30]. All included studies met the screening criteria of sample size > 500 and measured proteins > 50. Detailed information on the eight studies is summarized in Table S1. According to the consensus of the European Working Group on Sarcopenia in Older People (EWGSOP), muscle mass, muscle strength and physical function are three key indicators for diagnosing sarcopenia, which can be assessed respectively by fat‐free mass (FFM), grip strength and walking pace [3]. Therefore, nine sarcopenia‐related traits, including left arm FFM, left leg FFM, right arm FFM, right leg FFM, trunk FFM, whole body FFM, left hand grip strength, right hand grip strength and usual walking pace were considered as outcomes in this study. In Table S2, we presented the basic characteristics of these nine outcomes. Summary statistics for SNPs associated with muscle mass, including left arm FFM (*N* = 454 672), left leg FFM (*N* = 454 805), right arm FFM (*N* = 454 753), right leg FFM (*N* = 454 835), trunk FFM (*N* = 454 508) and whole body FFM (*N* = 454 850), were measured using bioelectrical impedance analysis (BIA) and extracted from the UKB [15, 31]. Summary statistics for SNPs associated with left hand grip strength (*N* = 461 026) and right hand grip strength (*N* = 461 089) were measured using the Jamar J00105 hydraulic hand dynamometer adjusted for hand size and extracted from the UKB [15, 31]. Additionally, GWAS summary statistics for SNPs associated with walking pace from the UKB involving 459 915 samples were also acquired. All datasets in this study were based on published research and public databases. Ethical approval and informed consent were obtained from the relevant institutions.

### 2.2 Selection of Instrument Variables

**FIGURE 1:**
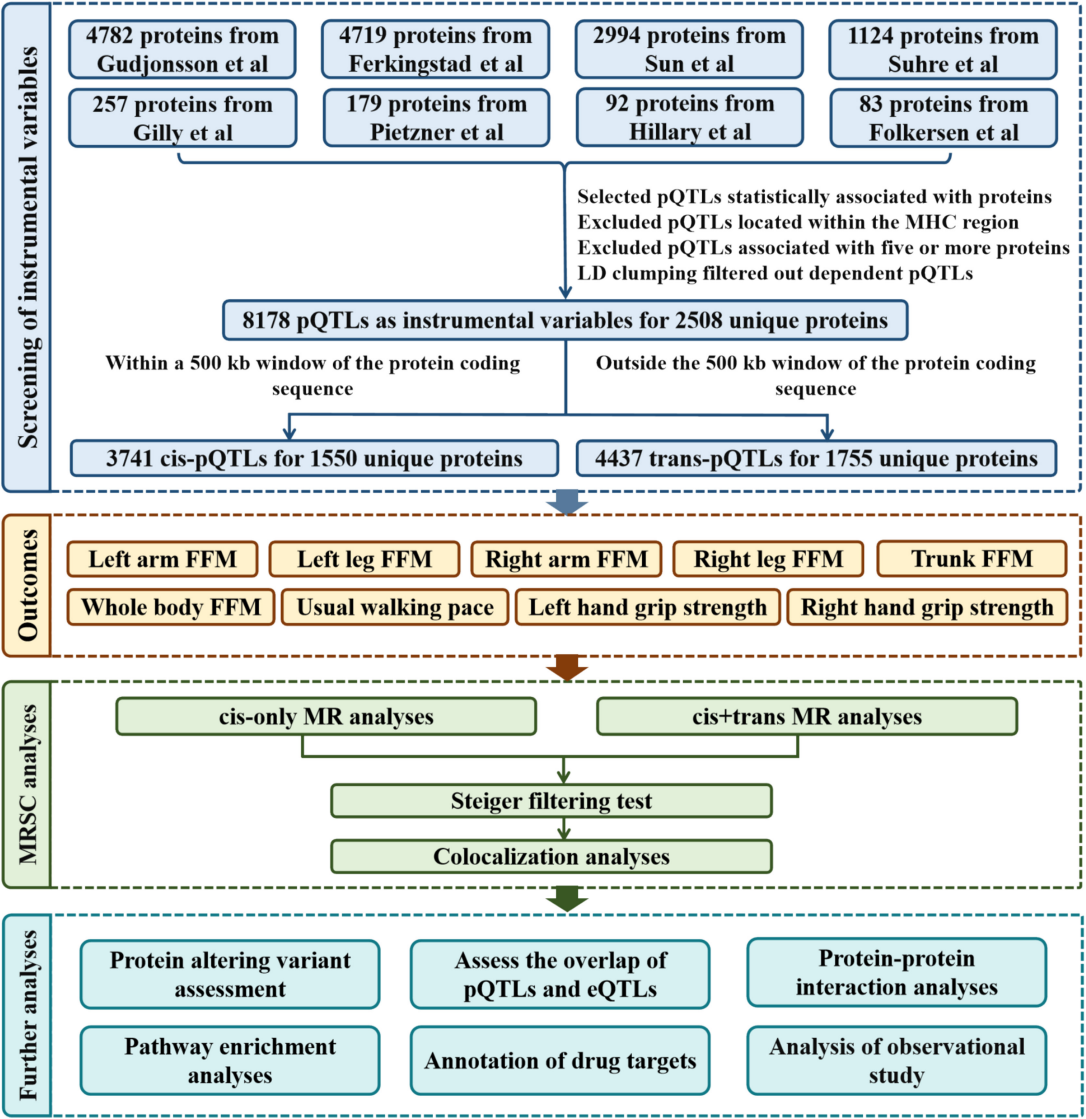
Study flow chart. eQTLs, expression quantitative trait loci; FFM, fat‐free mass; LD, linkage disequilibrium; MHC, major histocompatibility complex; MR, Mendelian randomization; pQTLs, protein quantitative trait loci.

The MR analysis conducted in this study employed pQTLs as instrumental variables (IVs) to establish causal relationships. As shown in Figure 1, the entire selection process of IVs was as follows: First, we utilized a threshold of *p* < 5 × 10^−8^, or the corresponding thresholds reported in related studies (as shown in Table S1), to select pQTLs. Second, we removed pQTLs located within the major histocompatibility complex region (Chromosome 6: 26–34 Mb). Third, pQTLs with high‐level pleiotropy associated with five or more proteins were excluded. Last, we performed linkage disequilibrium (LD) clumping to exclude dependent pQTLs within the corresponding region that were correlated with the most significant pQTL (upstream/downstream distance ≤ 5000 kb, *r* ^2^ > 0.01). For all pQTLs retained after screening, pQTLs within a 500‐kb window of the corresponding protein coding sequence were defined as cis‐pQTLs, whereas pQTLs outside the 500‐kb window of the protein coding sequence were defined as trans‐pQTLs.

### 2.3 MR Analyses

We conducted two‐sample MR analyses using genetically predicted circulating proteins as exposures and sarcopenia‐related traits as outcomes. The ‘TwoSampleMR’ R package (https://github.com/MRCIEU/TwoSampleMR) was utilized to perform cis‐only MR analyses and cis + trans MR analyses with cis‐pQTLs and cis + trans pQTLs as IVs, respectively, to identify causal proteins associated with the outcomes. When a single pQTL was used as an IV, the causal effect of the exposure on the outcome was estimated using the Wald ratio method. When multiple IVs were present, the causal inference was performed using the inverse variance–weighted (IVW) method [32]. In MR analysis, three core assumptions must be met to obtain valid results. Specifically, the genetic variant (or multiple genetic variants) used as IV for the risk factor must satisfy the following conditions: (1) reliably associate with the risk factor under investigation (relevance assumption), (2) not associate with any known or unknown confounding factors (independence assumption) and (3) influence the outcome only through the risk factor and not through any direct causal pathway (exclusion restriction assumption) [22]. In MR analysis, Assumption (3) could be violated by the presence of horizontal pleiotropy in IV, which may affect the reliability of MR results. Our study conducted sensitivity analyses to provide additional insights into the main MR results, using MR‐Egger regression to test and correct for directional horizontal pleiotropy among the IVs [33]. The MR‐Egger method is able to assess whether genetic variants have pleiotropic effects on the outcome that differ on average from zero (directional pleiotropy) [34]. Furthermore, when multiple IVs are used, heterogeneity in the estimates among different IVs can also lead to unreliable MR results. In sensitivity analyses, our study applied Cochran's *Q* test to identify heterogeneity and used the weighted median estimator (WME) [35] to correct for bias due to heterogeneity among the IVs (Cochran's *Q p* < 0.05). The WME method obtains the final causal effect estimate by weighting the effect estimates of each IV and calculating the weighted median. This approach effectively mitigates potential biases when some of the instruments are invalid, allowing WME to still provide accurate causal inferences [35]. Bonferroni correction was applied for multiple comparisons. The *p* value thresholds for cis‐only MR analyses and cis + trans MR analyses were provided in Table S3.

### 2.4 Steiger Filtering Test

The Steiger filtering test (using the ‘TwoSampleMR’ R package) was applied for the significant MR associations to determine the direction of causality and exclude reverse causality. If *p*_Steiger_ > 0.05, it is considered that there is no causality between the exposure and the outcome. If *p*_Steiger_ < 0.05, it is considered that there is a causal correlation between the exposure and the outcome, with ‘obverse’ representing a positive causal correlation, meaning the exposure leads to the outcome, whereas ‘reverse’ represents a reverse causal correlation.

### 2.5 Colocalization Analyses

To evaluate whether the circulating proteins and the sarcopenia‐related traits were affected by shared causal variants and to strengthen the evidence of causality, we utilized the ‘coloc’ R package (https://github.com/chr1swallace/coloc) to conduct original Bayesian colocalization analysis and the Sum of Single Effect (SuSiE) colocalization analysis separately on the significant associations prioritized by MR analyses and Steiger filtering tests. The SuSiE model is an improved approach that relaxes the single causal variant assumption of original Bayesian colocalization by aggregating single effects [36]. Therefore, when the assumption that there is only a single causal variant within the genetic region of interest was not met, we solely employed the SuSiE model to conduct colocalization analysis for each causal SNP individually and reported the maximum value of the posterior probability of hypothesis 4 (PPH4) in the supplementary tables. Ultimately, we integrated the outcomes of the original Bayesian colocalization analyses and the SuSiE colocalization analyses to report the final results. A PPH4 value exceeding 80% in either analysis was considered strong evidence of colocalization.

### 2.6 Protein‐Altering Variant (PAV) Assessment

PAVs are genetic variants capable of modifying the structure of proteins. These variations have the potential to alter the binding affinity between the protein itself and ligands in protein quantification assays, thereby potentially amplifying or attenuating the actual quantitative levels of the protein. In this study, we utilized the Ensembl Variant Effect Predictor (https://asia.ensembl.org/Tools/VEP) to annotate PAVs on all cis‐pQTLs prioritized by MRSC, as well as SNPs in LD (*r* ^2^ ≥ 0.8) with them. The variations labelled as PAVs include missense mutation, splice region variant, splice donor variant, splice acceptor variant, start loss, stop loss, stop gained, in‐frame insertion, in‐frame deletion and frameshift variant [21].

### 2.7 Overlap of pQTLs and Expression Quantitative Trait Loci (eQTLs)

The influence of genetic variations on protein levels is achieved through the regulation of the mRNA transcription process. In order to further enhance the biological interpretability of the causal associations identified in this study, we utilized the Genotype‐Tissue Expression (GTEx) Portal (https://www.gtexportal.org) to assess the overlap between pQTLs prioritized by MRSC (along with SNPs in LD with them) and their corresponding eQTLs. Additionally, we annotated those pQTLs that had significant corresponding eQTLs with concordant allelic directions.

### 2.8 Protein–Protein Interaction Analyses

Utilizing the Search Tool for the Retrieval of Interacting Genes (STRING, V11.5, https://string‐db.org/) platform, we conducted protein–protein interaction (PPI) analyses on proteins prioritized by cis‐only MRSC and cis + trans MRSC, respectively. A PPI enrichment *p* value of < 0.05 was considered to indicate that there are more interactions between the prioritized proteins than between a randomly selected set of proteins of the same size and degree distribution from the genome.

### 2.9 Pathway Enrichment Analyses

The ‘ClusterProfiler’ (https://github.com/YuLab‐SMU/clusterProfiler) R package and ‘ReactomePA’ (https://bioconductor.org/packages/release/bioc/html/ReactomePA.html) R package were utilized for conducting Reactome, Kyoto Encyclopedia of Genes and Genomes (KEGG) and Gene Ontology (GO) pathway enrichment analysis. Each pathway with a corrected *p* value of < 0.05 was considered significant.

### 2.10 Observational Study

By using data from 10 948 UKB participants, we conducted longitudinal linear regression analyses, to estimate the association between the levels of 2923 circulating proteins and the longitudinal changes in the nine sarcopenia‐related traits, adjusting for sex, age, body mass index (BMI), ethnicity and baseline levels of corresponding traits of sarcopenia. The nine sarcopenia‐related traits used in the observational study were completely consistent with the nine outcomes in the MR analysis. Bonferroni correction was used to control for multiple comparisons according to the number of proteins (0.05/2923).

### 2.11 Annotation of Drug Targets

The Therapeutic Target Database (TTD) [37] platform (https://db.idrblab.net/ttd/) was utilized to assess the druggability of the identified causal proteins, marking their target type, associated drugs and related diseases. Furthermore, we mapped these candidate proteins with the druggable genes reported by Finan et al. [38]. In this study, we applied the hierarchical method (described by Finan et al. in the paper) to annotate the priority tier for the druggable gene. Then, we also observed whether the protein product of the gene was targeted or predicted to be targeted by a small molecule/biotherapeutic.

## 3 Results

### 3.1 Screening of Instrumental Variables

Following a stepwise screening of pQTLs from eight studies, a total of 8178 pQTLs were retained as IVs for 4372 proteins (2508 unique proteins). The information for all IVs is presented in Table S4. Among the 8178 pQTLs, 3741 were identified as cis‐pQTLs for 2922 proteins (1550 unique proteins), whereas 4437 were classified as trans‐pQTLs for 2344 proteins (1755 unique proteins). In summary, a total of 4372 proteins with valid IVs were included in this study, of which 2028 proteins had only cis‐pQTLs as IVs, 1450 proteins had only trans‐pQTLs as IVs, and 894 proteins had both cis and trans instruments.

### 3.2 Evaluating the Causal Effect of Circulating Proteins on Sarcopenia‐Related Traits by MR Analyses

Compared to trans‐pQTLs, cis‐pQTLs were generally considered to directly regulate gene expression, exhibiting higher biological interpretability. In this study, we first performed cis‐only MR analyses using cis‐pQTLs as IVs. In the cis‐only MR analyses, 822 significant associations were identified and survived the Bonferroni correction (Table S5). Associations of 211 cis‐only MR‐prioritized proteins (155 unique proteins) with sarcopenia‐related traits were summarized in Table S6. According to the Bonferroni‐corrected *p* values in the MR analyses, the sensitivity cis‐only MR analyses (Table S7) discovered 24 new causal associations for five proteins (Table S8).

In the MR analyses, incorporating trans‐pQTLs as IVs may strengthen the reliability of the protein–trait associations. Therefore, we further conducted cis + trans MR analyses using all (cis + trans) pQTLs as IVs. In the cis + trans MR analyses, 1004 protein–trait causal associations were discovered (Table S9). Associations of 300 cis + trans MR–prioritized proteins (247 unique proteins) with 9 sarcopenia‐related traits were summarized in Table S10. As shown in Tables S11 and S12, 86 causal associations were identified by the sensitivity cis + trans MR analyses, with 85 being newly discovered.

### 3.3 Determining the Causal Direction by Steiger Filtering Test

To determine the direction of causality and exclude reverse causality, we performed Steiger filtering tests on significant causal associations prioritized by cis‐only MR analyses and cis + trans MR analyses, respectively (Tables S13 and S14). As a result, only one protein–trait association prioritized by cis‐only MR analyses and two associations prioritized by cis + trans MR analyses were filtered out through the Steiger filtering tests (Steiger *p* value of > 0.05). All significant associations demonstrated an obverse causal direction (from proteins to sarcopenia‐related traits).

### 3.4 Colocalization Analysis

We conducted original Bayesian colocalization analyses (applicable when there is only one causal SNP per trait) and colocalization using the SuSiE regression model, respectively. The PPH4 value was considered strong evidence for colocalization if it exceeded 0.8 in either of the two analyses. Among the 821 causal associations prioritized by cis‐only MR analyses and Steiger filtering tests, 181 of them exhibited strong evidence of colocalization (Table S15). For 1002 associations prioritized by cis + trans MR analyses and Steiger filtering tests (Table S16), 227 associations had strong evidence of colocalization.

**FIGURE 2:**
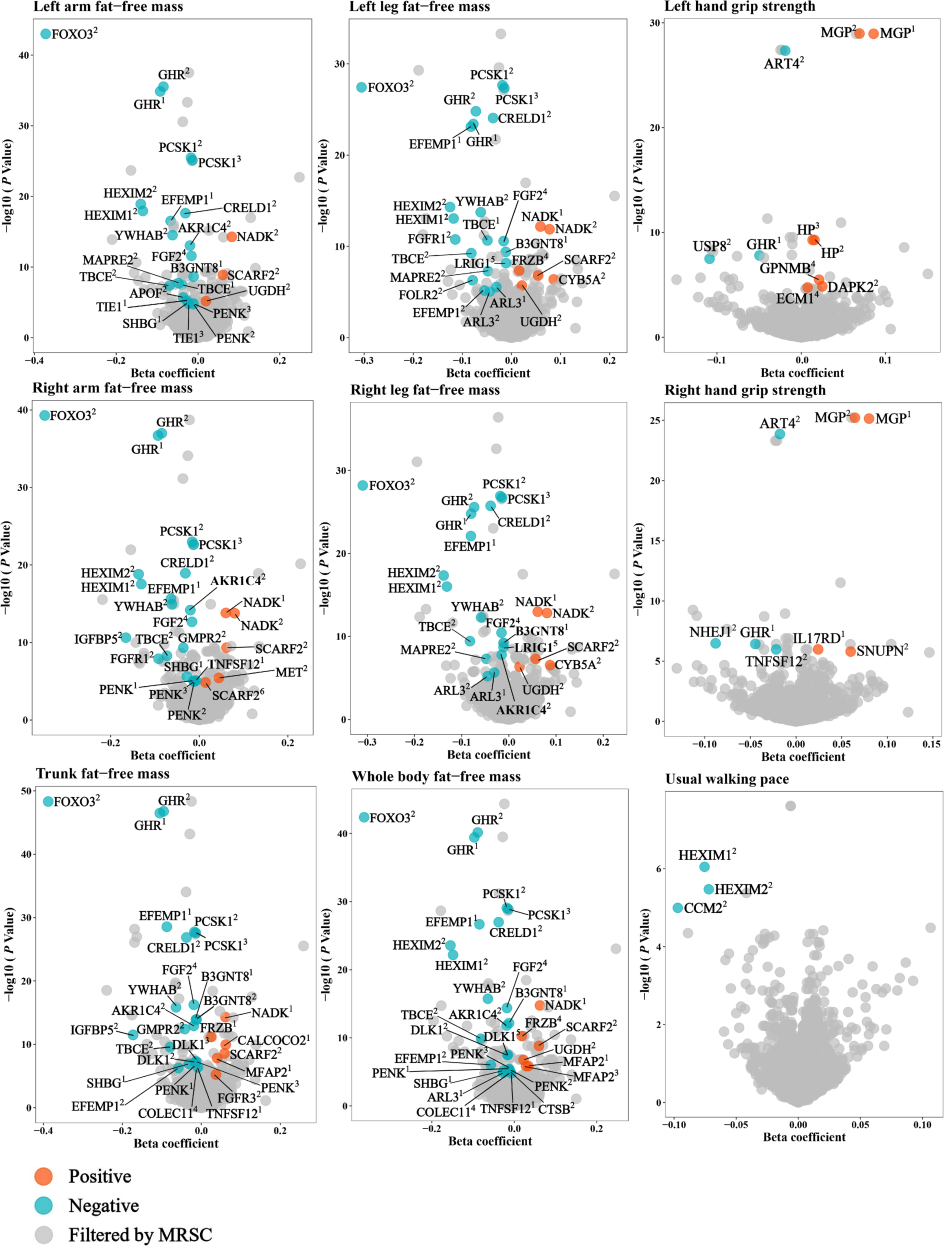
Volcano plot for results of the cis‐only MRSC analyses. Sources of the instruments: 1 represents Gudjonsson et al.; 2 represents Ferkingstad et al.; 3 represents Sun et al.; 4 represents Suhre et al.; 5 represents Gilly et al.; 6 represents Hillary et al. MRSC, a sequential stepwise analysis of Mendelian randomization, Steiger filtering test and colocalization.

**FIGURE 3:**
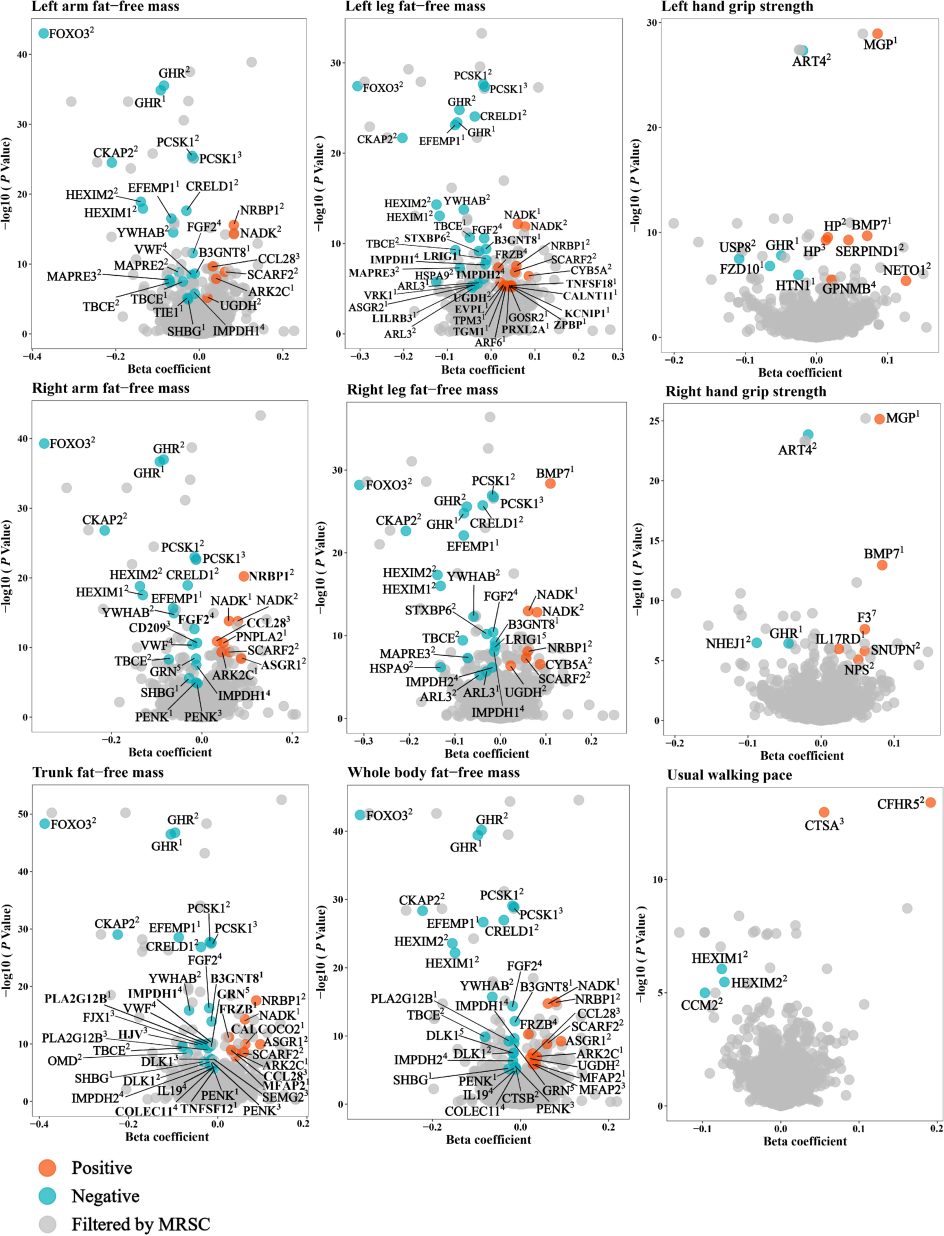
Volcano plot for results of the cis + trans MRSC analyses. Sources of the instruments: 1 represents Gudjonsson et al.; 2 represents Ferkingstad et al.; 3 represents Sun et al; 4 represents Suhre et al.; 5 represents Gilly et al.; 7 represents Folkersen et al. MRSC, a sequential stepwise analysis of Mendelian randomization, Steiger filtering test and colocalization.

In summary, a total of 181 causal associations for 65 proteins (47 unique proteins) were prioritized by the cis‐only MRSC stepwise screening analyses (Table 1, Figure 2), and 227 associations for 91 proteins (79 unique proteins) were prioritized by the cis + trans MRSC analyses (Table 2, Figure 3). Despite varying exposure sources and outcomes, each identified protein exhibited a consistent direction of correlation. This consistency reflects the reliability of our research method and suggests the presence of similar pathological mechanisms among the nine traits of sarcopenia.

**TABLE 1:** Protein–phenotype associations prioritized by MRSC using cis‐pQTLs.

Uniprot	Protein	Outcome	Direction
O43508^a^, ^b^, ^m^	Tumour necrosis factor ligand superfamily member 12 (TNFSF12)	Right arm FFM^h^, trunk FFM^h^, whole body FFM^h^ and right hand grip strength^g^	Negative
O43524^b^	Forkhead box protein O3 (FOXO3)	Left arm FFM, left leg FFM, right arm FFM, right leg FFM and whole body FFM	Negative
O60701^b^	UDP‐glucose 6‐dehydrogenase (UGDH)	Left arm FFM, left leg FFM, right leg FFM and whole body FFM	Positive
O94992^b^	Protein HEXIM1 (HEXIM1)	Left arm FFM, left leg FFM, usual walking pace, right arm FFM, right leg FFM and whole body FFM	Negative
O95149^b^	Snurportin‐1 (SNUPN)	Right hand grip strength	Positive
O95544^a^, ^b^, ^m^	NAD kinase (NADK)	Left arm FFM^g^, left leg FFM, right arm FFM, right leg FFM, trunk FFM^h^ and whole body FFM^h^	Positive
P00167^b^, ^m^	Cytochrome b5 (CYB5A)	Left leg FFM and right leg FFM	Positive
P00738^b^, ^c^	Haptoglobin (HP)	Left hand grip strength	Positive
P01210^a^, ^b^, ^c^	Proenkephalin‐A (PENK)	Left arm FFM^g^, right arm FFM, trunk FFM^h^ and whole body FFM	Negative
P04278^a^	Sex hormone‐binding globulin (SHBG)	Left arm FFM, right arm FFM, trunk FFM and whole body FFM	Negative
P07858^b^	Cathepsin B (CTSB)	Whole body FFM	Negative
P08493^a^, ^b^, ^m^	Matrix Gla protein (MGP)	Left hand grip strength and right hand grip strength	Positive
P08581^b^, ^m^	Hepatocyte growth factor receptor (MET)	Right arm FFM	Positive
P09038^d^	Fibroblast growth factor 2 (FGF2)	Left arm FFM, left leg FFM, right arm FFM, right leg FFM, trunk FFM and whole body FFM	Negative
P10912^a^, ^b^	Growth hormone receptor (GHR)	Left arm FFM, left leg FFM, right arm FFM, right leg FFM, trunk FFM, whole body FFM, left hand grip strength^h^ and right hand grip strength^h^	Negative
P11362^b^	Fibroblast growth factor receptor 1 (FGFR1)	Left leg FFM and right arm FFM	Negative
P14207^b^	Folate receptor beta (FOLR2)	Left leg FFM	Negative
P17516^b^, ^m^	Aldo‐keto reductase family 1 member C4 (AKR1C4)	Left arm FFM, right arm FFM, right leg FFM, trunk FFM and whole body FFM	Negative
P22607^b^, ^m^	Fibroblast growth factor receptor 3 (FGFR3)	Trunk FFM	Positive
P24593^b^	Insulin‐like growth factor‐binding protein 5 (IGFBP5)	Right arm FFM and trunk FFM	Negative
P29120^b^, ^c^, ^m^	Neuroendocrine convertase 1 (PCSK1)	Left arm FFM, left leg FFM, right arm FFM, right leg FFM, trunk FFM and whole body FFM	Negative
P31946^b^, ^m^	14‐3‐3 Protein beta/alpha (YWHAB)	Left arm FFM, left leg FFM, right arm FFM, right leg FFM, trunk FFM and whole body FFM	Negative
P35590^a^, ^c^, ^m^	Tyrosine–protein kinase receptor Tie‐1 (TIE1)	Left arm FFM	Negative
P36405^a^, ^b^	ADP‐ribosylation factor‐like protein 3 (ARL3)	Left leg FFM, right leg FFM and whole body FFM^h^	Negative
P40818^b^, ^m^	Ubiquitin carboxyl‐terminal hydrolase 8 (USP8)	Left hand grip strength	Negative
P55001^a^, ^c^, ^m^	Microfibrillar‐associated protein 2 (MFAP2)	Trunk FFM^i^ and whole body FFM	Positive
P80370^b^, ^c^, ^e^	Protein delta homologue 1 (DLK1)	Trunk FFM^j^ and whole body FFM^i^	Negative
Q12805^a^, ^b^	EGF‐containing fibulin‐like extracellular matrix protein 1 (EFEMP1)	Left arm FFM^h^, left leg FFM, right arm FFM^h^, right leg FFM^h^, trunk FFM and whole body FFM	Negative
Q13137^a^, ^m^	Calcium‐binding and coiled‐coil domain‐containing protein 2 (CALCOCO2)	Trunk FFM	Positive
Q13790^b^, ^m^	Apolipoprotein F (APOF)	Left arm FFM	Negative
Q14956^d^	Transmembrane glycoprotein NMB (GPNMB)	Left hand grip strength	Positive
Q15555^b^	Microtubule‐associated protein RP/EB family member 2 (MAPRE2)	Left arm FFM, left leg FFM and right leg FFM	Negative
Q15813^a^, ^b^	Tubulin‐specific chaperone E (TBCE)	Left arm FFM, left leg FFM, right arm FFM^g^, right leg FFM^g^, trunk FFM^g^ and whole body FFM^g^	Negative
Q16610^d^, ^m^	Extracellular matrix protein 1 (ECM1)	Left hand grip strength	Positive
Q7Z7M8^a^, ^b^, ^m^	UDP‐GlcNAc:betaGal beta‐1,3‐N‐acetylglucosaminyltransferase 8 (B3GNT8)	Left arm FFM^h^, left leg FFM^h^, right leg FFM^h^, trunk FFM and whole body FFM^h^	Negative
Q8NFM7^a^, ^m^	Interleukin‐17 receptor D (IL17RD)	Right hand grip strength	Positive
Q92765^a^, ^d^, ^m^	Secreted frizzled‐related protein 3 (FRZB)	Left leg FFM^g^, whole body FFM^g^ and trunk FFM^l^	Positive
Q93070^b^, ^m^	Ecto‐ADP‐ribosyltransferase 4 (ART4)	Left hand grip strength and right hand grip strength	Negative
Q96GP6^b^, ^f^, ^m^	Scavenger receptor class F member 2 (SCARF2)	Left arm FFM^k^, left leg FFM^k^, right arm FFM, right leg FFM^k^, trunk FFM^k^ and whole body FFM^k^	Positive
Q96HD1^b^	Protein disulfide isomerase CRELD1 (CRELD1)	Left arm FFM, left leg FFM, right arm FFM, right leg FFM, trunk FFM and whole body FFM	Negative
Q96JA1^e^, ^m^	Leucine‐rich repeats and immunoglobulin‐like domains protein 1 (LRIG1)	Left leg FFM and right leg FFM	Negative
Q96MH2^b^	Protein HEXIM2 (HEXIM2)	Left arm FFM, left leg FFM, right arm FFM, right leg FFM, whole body FFM and usual walking pace	Negative
Q9BSQ5^b^, ^m^	Cerebral cavernous malformations 2 protein (CCM2)	Usual walking pace	Negative
Q9BWP8^d^	Collectin‐11 (COLEC11)	Trunk FFM and whole body FFM	Negative
Q9H9Q4^b^	Nonhomologous end‐joining factor 1 (NHEJ1)	Right hand grip strength	Negative
Q9P2T1^b^, ^m^	GMP reductase 2 (GMPR2)	Right arm FFM and trunk FFM	Negative
Q9UIK4^b^	Death‐associated protein kinase 2 (DAPK2)	Left hand grip strength	Positive

Abbreviations: FFM, fat‐free mass; MR, Mendelian randomization; MRSC, a sequential stepwise analysis of MR, Steiger filtering test and colocalization; pQTLs, protein quantitative trait loci.

^a^ Represents Gudjonsson et al.

^b^ Represents Ferkingstad et al.

^c^ Represents Sun et al.

^d^ Represents Suhre et al.

^e^ Represents Gilly et al.

^f^ Represents Hillary et al.

^g^ Significant association was not found by using instruments from Gudjonsson et al.

^h^ Significant association was not found by using instruments from Ferkingstad et al.

^i^ Significant association was not found by using instruments from Sun et al.

^j^ Significant association was not found by using instruments from Gilly et al.

^k^ Significant association was not found by using instruments from Hillary et al.

^l^ Significant association was not found by using instruments from Suhre et al.

^m^ Protein‐altering variants were used as instrumental variables.

**TABLE 2:** Protein–phenotype associations prioritized by MRSC using all pQTLs.

Uniprot	Protein	Outcome	Direction
O14653^a^	Golgi SNAP receptor complex member 2 (GOSR2)	Left leg FFM	Positive
O43508^a^, ^l^	Tumour necrosis factor ligand superfamily member 12 (TNFSF12)	Trunk FFM	Negative
O43524^b^	Forkhead box protein O3 (FOXO3)	Left arm FFM, left leg FFM, right arm FFM, right leg FFM and whole body FFM	Negative
O60701^b^	UDP‐glucose 6‐dehydrogenase (UGDH)	Left arm FFM, left leg FFM, right leg FFM and whole body FFM	Positive
O75022^a^	Leukocyte immunoglobulin‐like receptor subfamily B member 3 (LILRB3)	Left leg FFM	Negative
O94992^b^	Protein HEXIM1 (HEXIM1)	Left arm FFM, left leg FFM, right arm FFM, right leg FFM, whole body FFM and usual walking pace	Negative
O95149^b^	Snurportin‐1 (SNUPN)	Right hand grip strength	Positive
O95544^a^, ^b^, ^l^	NAD kinase (NADK)	Left arm FFM^g^, left leg FFM, right arm FFM, right leg FFM, trunk FFM^h^ and whole body FFM^h^	Positive
P00167^b^, ^l^	Cytochrome b5 (CYB5A)	Left leg FFM and right leg FFM	Positive
P00738^b^, ^c^	Haptoglobin (HP)	Left hand grip strength	Positive
P01210^a^, ^c^	Proenkephalin‐A (PENK)	Right arm FFM, trunk FFM and whole body FFM	Negative
P04275^d^	von Willebrand factor (VWF)	Left arm FFM, right arm FFM and trunk FFM	Negative
P04278^a^	Sex hormone‐binding globulin (SHBG)	Left arm FFM, right arm FFM, trunk FFM and whole body FFM	Negative
P05546^b^	Heparin cofactor 2 (SERPIND1)	Left hand grip strength	Positive
P06753^a^	Tropomyosin alpha‐3 chain (TPM3)	Left leg FFM	Positive
P07306^b^	Asialoglycoprotein receptor 1 (ASGR1)	Left arm FFM, trunk FFM and whole body FFM	Positive
P07307^a^	Asialoglycoprotein receptor 2 (ASGR2)	Left leg FFM	Negative
P07858^b^	Cathepsin B (CTSB)	Whole body FFM	Negative
P08493^a^, ^l^	Matrix Gla protein (MGP)	Left hand grip strength and right hand grip strength	Positive
P09038^d^	Fibroblast growth factor 2 (FGF2)	Left arm FFM, left leg FFM, right arm FFM, right leg FFM, trunk FFM and whole body FFM	Negative
P0C0P6^b^	Neuropeptide S (NPS)	Right hand grip strength	Positive
P10619^c^	Lysosomal protective protein (CTSA)	Usual walking pace	Positive
P10912^a^, ^b^	Growth hormone receptor (GHR)	Left arm FFM, left leg FFM, right arm FFM, right leg FFM, trunk FFM, whole body FFM, left hand grip strength^h^ and right hand grip strength^h^	Negative
P12268^d^	Inosine‐5′‐monophosphate dehydrogenase 2 (IMPDH2)	Left leg FFM, right leg FFM, trunk FFM and whole body FFM	Negative
P13726^f^	Tissue factor (F3)	Right hand grip strength	Positive
P15515^a^	Histatin‐1 (HTN1)	Left hand grip strength	Negative
P18075^a^	Bone morphogenetic protein 7 (BMP7)	Right leg FFM, left hand grip strength and right hand grip strength	Positive
P20839^d^	Inosine‐5′‐monophosphate dehydrogenase 1 (IMPDH1)	Left arm FFM, left leg FFM, right arm FFM, right leg FFM, trunk FFM and whole body FFM	Negative
P22735^a^	Protein–glutamine gamma‐glutamyltransferase K (TGM1)	Left leg FFM	Positive
P28799^e^	Progranulin (GRN)	Right arm FFM, trunk FFM and whole body FFM	Negative
P29120^b^, ^c^, ^l^	Neuroendocrine convertase 1 (PCSK1)	Left arm FFM, left leg FFM, right arm FFM, right leg FFM, trunk FFM and whole body FFM	Negative
P31946^b^, ^l^	14–3‐3 protein beta/alpha (YWHAB)	Left arm FFM, left leg FFM, right arm FFM, right leg FFM, trunk FFM and whole body FFM	Negative
P35590^a^, ^l^	Tyrosine‐protein kinase receptor Tie‐1 (TIE1)	Left arm FFM	Negative
P36405^a^, ^b^	ADP‐ribosylation factor‐like protein 3 (ARL3)	Left leg FFM and right leg FFM	Negative
P38646^b^	Stress‐70 protein, mitochondrial (HSPA9)	Left leg FFM and right leg FFM	Negative
P40818^b^, ^l^	Ubiquitin carboxyl‐terminal hydrolase 8 (USP8)	Left hand grip strength	Negative
P55001^a^, ^c^, ^l^	Microfibrillar‐associated protein 2 (MFAP2)	Trunk FFM^i^ and whole body FFM	Positive
P62330^a^	ADP‐ribosylation factor 6 (ARF6)	Left leg FFM	Positive
P80370^b^, ^c^, ^e^	Protein delta homologue 1 (DLK1)	Trunk FFM^j^ and whole body FFM^i^	Negative
Q02383^c^	Semenogelin‐2 (SEMG2)	Trunk FFM	Negative
Q12805^a^	EGF‐containing fibulin‐like extracellular matrix protein 1 (EFEMP1)	Left arm FFM, left leg FFM, right arm FFM, right leg FFM, trunk FFM and whole body FFM	Negative
Q13137^a^, ^l^	Calcium‐binding and coiled‐coil domain‐containing protein 2 (CALCOCO2)	Trunk FFM	Positive
Q14956^d^	Transmembrane glycoprotein NMB (GPNMB)	Left hand grip strength	Positive
Q15555^b^	Microtubule‐associated protein RP/EB family member 2 (MAPRE2)	Left arm FFM	Negative
Q15813^a^, ^b^	Tubulin‐specific chaperone E (TBCE)	Left arm FFM, left leg FFM, right arm FFM^g^, right leg FFM^g^, trunk FFM^g^ and whole body FFM^g^	Negative
Q6ZSG1^a^	E3 ubiquitin‐protein ligase ARK2C (ARK2C)	Left arm FFM, right arm FFM, trunk FFM and whole body FFM	Positive
Q6ZVN8^c^	Hemojuvelin (HJV)	Trunk FFM	Negative
Q7Z7M8^a^, ^l^	UDP‐GlcNAc:betaGal beta‐1,3‐N‐acetylglucosaminyltransferase 8 (B3GNT8)	Left arm FFM, left leg FFM, right leg FFM, trunk FFM and whole body FFM	Negative
Q86VR8^c^	Four‐jointed box protein 1 (FJX1)	Trunk FFM	Negative
Q8NCW6^a^	Polypeptide N‐acetylgalactosaminyltransferase 11 (GALNT11)	Left leg FFM	Positive
Q8NFM7^a^, ^l^	Interleukin‐17 receptor D (IL17RD)	Right hand grip strength	Positive
Q8NFX7^b^	Syntaxin‐binding protein 6 (STXBP6)	Left leg FFM and right leg FFM	Negative
Q8TDF5^b^	Neuropilin and tolloid‐like protein 1 (NETO1)	Left hand grip strength	Positive
Q8WWK9^b^	Cytoskeleton‐associated protein 2 (CKAP2)	Left arm FFM, left leg FFM, right arm FFM, right leg FFM, trunk FFM and whole body FFM	Negative
Q92765^a^, ^d^, ^l^	Secreted frizzled‐related protein 3 (FRZB)	Trunk FFM^k^, left leg FFM^g^ and whole body FFM^g^	Positive
Q92817^a^	Envoplakin (EVPL)	Left leg FFM	Positive
Q93070^b^, ^l^	Ecto‐ADP‐ribosyltransferase 4 (ART4)	Left hand grip strength and right hand grip strength	Negative
Q96AD5^a^	Patatin‐like phospholipase domain‐containing protein 2 (PNPLA2)	Right arm FFM	Positive
Q96GP6^b^, ^l^	Scavenger receptor class F member 2 (SCARF2)	Left arm FFM, left leg FFM, right arm FFM, right leg FFM, trunk FFM and whole body FFM	Positive
Q96HD1^b^	Protein disulfide isomerase CRELD1 (CRELD1)	Left arm FFM, left leg FFM, right arm FFM, right leg FFM, trunk FFM and whole body FFM	Negative
Q96JA1^e^, ^l^	Leucine‐rich repeats and immunoglobulin‐like domains protein 1 (LRIG1)	Left leg FFM and right leg FFM	Negative
Q96MH2^b^	Protein HEXIM2 (HEXIM2)	Left arm FFM, left leg FFM, right arm FFM, right leg FFM, whole body FFM and usual walking pace	Negative
Q99983^b^	Osteomodulin (OMD)	Trunk FFM	Negative
Q99986^a^	Serine/threonine–protein kinase VRK1 (VRK1)	Left leg FFM	Negative
Q9BRX8^a^	Peroxiredoxin‐like 2A (PRXL2A)	Left leg FFM	Positive
Q9BS86^a^	Zona pellucida‐binding protein 1 (ZPBP)	Left leg FFM	Positive
Q9BSQ5^b^, ^l^	Cerebral cavernous malformations 2 protein (CCM2)	Usual walking pace	Negative
Q9BWP8^d^	Collectin‐11 (COLEC11)	Trunk FFM and whole body FFM	Negative
Q9BX93^a^, ^c^	Group XIIB secretory phospholipase A2‐like protein (PLA2G12B)	Trunk FFM and whole body FFM^i^	Negative
Q9BXR6^b^	Complement factor H‐related protein 5 (CFHR5)	Usual walking pace	Positive
Q9H9Q4^b^	Nonhomologous end‐joining factor 1 (NHEJ1)	Right hand grip strength	Negative
Q9NNX6^c^	CD209 antigen (CD209)	Right arm FFM	Negative
Q9NRJ3^c^	C–C motif chemokine 28 (CCL28)	Left arm FFM, right arm FFM, trunk FFM and whole body FFM	Positive
Q9NZI2^a^	Kv channel‐interacting protein 1 (KCNIP1)	Left leg FFM	Positive
Q9UHD0^d^	Interleukin‐19 (IL19)	Trunk FFM and whole body FFM	Negative
Q9UHY1^b^	Nuclear receptor‐binding protein (NRBP1)	Left arm FFM, left leg FFM, right arm FFM, right leg FFM, trunk FFM and whole body FFM	Positive
Q9ULW2^a^	Frizzled‐10 (FZD10)	Left hand grip strength	Negative
Q9UNG2^a^	Tumour necrosis factor ligand superfamily member 18 (TNFSF18)	Left leg FFM	Positive
Q9UPY8^b^	Microtubule‐associated protein RP/EB family member 3 (MAPRE3)	Left arm FFM, left leg FFM and right leg FFM	Negative

Abbreviations: FFM, fat‐free mass; MR, Mendelian randomization; MRSC, a sequential stepwise analysis of MR, Steiger filtering test and colocalization; pQTLs, protein quantitative trait loci.

^a^ Represents Gudjonsson et al.

^b^ Represents Ferkingstad et al.

^c^ Represents Sun et al.

^d^ Represents Suhre et al.

^e^ Represents Gilly et al.

^f^ Represents Folkersen et al.

^g^ Significant association was not found by using instruments from Gudjonsson et al.

^h^ Significant association was not found by using instruments from Ferkingstad et al.

^i^ Significant association was not found by using instruments from Sun et al.

^j^ Significant association was not found by using instruments from Gilly et al.

^k^ Significant association was not found by using instruments from Suhre et al.

^l^ Protein‐altering variants were used as cis‐pQTLs.

### 3.5 PAV Assessments on the cis‐pQTLs

PAV may alter the affinity of the adapter by affecting the conformational structure of the protein it encodes, ultimately leading to measurement artefacts. Therefore, we performed PAV assessments on all cis‐IVs prioritized by MRSC, as well as SNPs in LD (*r* ^2^ ≥ 0.8) with them. We annotated the causal proteins with PAVs as IVs in Tables 1, 2 and S17, respectively. Among the 60 cis‐IVs for 65 proteins, 34 (36 proteins) were non‐PAVs, and 26 (30 proteins) were either PAVs or in LD with PAVs. Notably, the IVs for APOF included one PAV and one non‐PAV.

### 3.6 Evaluating the Overlap of pQTLs and eQTLs

In order to enhance the biological interpretability of significant associations identified by MRSC, we evaluated the association between pQTLs and proteins as being driven by transcriptional levels, rather than other mechanisms, by analysing the overlap of pQTLs and eQTLs. We annotated those pQTLs that had significant corresponding eQTLs in at least one tissue with concordant allelic directions, as well as SNPs in LD (*r* ^2^ ≥ 0.8) with them. Among the 67 cis‐pQTLs prioritized by cis‐only MRSC analyses (Table S18), 54 overlapped with the corresponding eQTLs that have consistent genetic effect directions in at least one tissue. For 96 pQTLs prioritized by cis + trans MRSC analyses including 48 cis‐pQTLs and 48 trans‐pQTLs (Table S19), 42 of them (41 cis‐pQTLs and one trans‐pQTL) had significant corresponding eQTLs in at least one tissue with concordant allelic directions.

### 3.7 PPI Analyses of the MRSC Prioritized Proteins

We respectively conducted PPI analyses for cis‐only MRSC and cis + trans MRSC prioritized proteins to analyse their interactions. In the PPI network of the proteins prioritized by cis‐only MRSC (Figure S1A), by using the interaction score threshold of 0.4 with medium confidence, the STRING PPI analysis yielded a clustered network with a clustering coefficient of 0.323. The PPI network contained 47 nodes with 24 edges (11 expected edges), indicating this network has significantly more interactions than expected (enrichment *P* = 0.000404). Figure S1B presented the PPI network of the proteins prioritized by cis + trans MRSC, the STRING PPI analysis yielded a clustered network with a clustering coefficient of 0.355. The PPI network contained 79 nodes with 50 edges (28 expected edges), demonstrating that the network has significantly more interactions than expected (enrichment *P* = 8.76 × 10^−5^).

### 3.8 Pathway Enrichment Analyses

To investigate the enriched functions of candidate causal proteins and the potential pathogenic mechanisms of sarcopenia, we performed Reactome, KEGG and GO pathway enrichment analyses on proteins prioritized by cis‐only MRSC and cis + trans MRSC analyses. For proteins prioritized by cis‐only MRSC analyses, Reactome (Table S20, Figure S2A), KEGG (Table S21, Figure S3A) and GO (Table S22, Figure S4A) pathway enrichment analyses identified 52, 12 and 79 enriched pathways (adjusted *P* < 0.05), respectively.

For proteins identified by cis + trans MRSC analyses, 15 significant pathways were enriched through the GO (Table S23, Figure S4B) pathway enrichment analyses. However, neither Reactome nor KEGG analyses identified any enriched pathways for these cis + trans prioritized proteins.

### 3.9 Observational Study

We conducted longitudinal linear regression analyses based on the UKB datasets to explore more potential biomarkers for sarcopenia. A total of 492 significant associations for 197 unique proteins were identified through linear regression (Table S24). The 197 proteins and their associated traits are summarized in Table S25. Of the 2923 proteins from UKB, 45 of them were overlapped with proteins prioritized by MRSC. As shown in Table S26, we compared the results of 28 overlapped proteins that were prioritized by cis‐only MRSC. We observed that only one protein–trait [cathepsin B (CTSB)–whole body FFM] association was found to be significant in the linear regression, with the direction being consistent with the MR result. In Table S27, we summarized the comparisons of 38 overlapped proteins that were prioritized by cis + trans MRSC. Longitudinal linear regression analyses determined three significant associations consistent with the direction of MR, which corresponded to two proteins, including Asialoglycoprotein receptor 1 (ASGR1) and CTSB.

### 3.10 Annotation of Drug Targets

This study identified 89 causal proteins as potential pathogenic proteins for sarcopenia through MRSC analyses. In Table S28, we summarized the drug target annotations for the 89 proteins based on evidence provided by Finan et al. and the TTD platform. According to the classification of druggable genes by Finan et al., out of the 89 proteins, 47 were reported as druggable targets. Evidence from the TTD indicated that among the 89 causal proteins, 32 were identified as existing or potential drug targets. Regarding the 32 proteins with database evidence supporting them as drug targets, 27 of them (84%) overlapped with the druggable gene set provided by Finan et al. For those proteins without database evidence support, less than half of the proteins (20/57) were found to overlap with the druggable gene set. Table 3 presented all the annotated drug target proteins among the 89 MRSC‐prioritized proteins.

**TABLE 3:** List of the MRSC prioritized proteins that were drug targets or to be druggable.

Uniprot	Protein	Druggability tier^a^	Target type^b^
O43508	Tumour necrosis factor ligand superfamily member 12 (TNFSF12)	Tier 1	Clinical trial target
O75022	Leukocyte immunoglobulin‐like receptor subfamily B member 3 (LILRB3)	/	Literature‐reported target
O94992	Protein HEXIM1 (HEXIM1)	/	Literature‐reported target
P00738	Haptoglobin (HP)	Tier 3A	Clinical trial target
P01210	Proenkephalin‐A (PENK)	Tier 3A	/
P04275	von Willebrand factor (VWF)	Tier 1	Successful target
P04278	Sex hormone‐binding globulin (SHBG)	Tier 1	/
P05546	Heparin cofactor 2 (SERPIND1)	Tier 3B	Literature‐reported target
P07306	Asialoglycoprotein receptor 1 (ASGR1)	Tier 3A	Clinical trial target
P07858	Cathepsin B (CTSB)	Tier 1	Preclinical target
P08493	Matrix Gla protein (MGP)	Tier 3B	/
P08581	Hepatocyte growth factor receptor (MET)	Tier 1	Successful target
P09038	Fibroblast growth factor 2 (FGF2)	Tier 1	Successful target
P0C0P6	Neuropeptide S (NPS)	Tier 3B	/
P10619	Lysosomal protective protein (CTSA)	Tier 2	Clinical trial target
P10912	Growth hormone receptor (GHR)	Tier 1	Successful target
P11362	Fibroblast growth factor receptor 1 (FGFR1)	Tier 1	Successful target
P12268	Inosine‐5′‐monophosphate dehydrogenase 2 (IMPDH2)	Tier 1	Successful target
P13726	Tissue factor (F3)	Tier 1	Successful target
P14207	Folate receptor beta (FOLR2)	Tier 3B	Literature‐reported target
P15515	Histatin‐1 (HTN1)	Tier 3B	/
P18075	Bone morphogenetic protein 7 (BMP7)	Tier 3B	Clinical trial target
P20839	Inosine‐5′‐monophosphate dehydrogenase 1 (IMPDH1)	Tier 1	Successful target
P22607	Fibroblast growth factor receptor 3 (FGFR3)	Tier 1	Successful target
P22735	Protein–glutamine gamma–glutamyltransferase K (TGM1)	Tier 2	Clinical trial target
P24593	Insulin‐like growth factor‐binding protein 5 (IGFBP5)	Tier 2	Literature‐reported target
P28799	Progranulin (GRN)	Tier 3A	Literature‐reported target
P29120	Neuroendocrine convertase 1 (PCSK1)	Tier 3A	Literature‐reported target
P35590	Tyrosine–protein kinase receptor Tie‐1 (TIE1)	Tier 1	Literature‐reported target
P38646	Stress‐70 protein, mitochondrial (HSPA9)	/	Clinical trial target
P40818	Ubiquitin carboxyl‐terminal hydrolase 8 (USP8)	/	Preclinical target
P55001	Microfibrillar‐associated protein 2 (MFAP2)	Tier 3A	/
P80370	Protein delta homologue 1 (DLK1)	Tier 1	Clinical trial target
Q02383	Semenogelin‐2 (SEMG2)	Tier 3B	/
Q12805	EGF‐containing fibulin‐like extracellular matrix protein 1 (EFEMP1)	Tier 3A	/
Q13790	Apolipoprotein F (APOF)	Tier 3A	/
Q14956	Transmembrane glycoprotein NMB (GPNMB)	Tier 1	Literature‐reported target
Q16610	Extracellular matrix protein 1 (ECM1)	Tier 3B	/
Q86VR8	Four‐jointed box protein 1 (FJX1)	Tier 3B	/
Q8NFM7	Interleukin‐17 receptor D (IL17RD)	/	Successful target
Q92765	Secreted frizzled‐related protein 3 (FRZB)	Tier 3A	/
Q93070	Ecto‐ADP‐ribosyltransferase 4 (ART4)	Tier 3B	/
Q99983	Osteomodulin (OMD)	Tier 3B	/
Q99986	Serine/threonine–protein kinase VRK1 (VRK1)	Tier 3A	/
Q9BX93	Group XIIB secretory phospholipase A2‐like protein (PLA2G12B)	Tier 3B	/
Q9BXR6	Complement factor H‐related protein 5 (CFHR5)	Tier 3A	/
Q9NNX6	CD209 antigen (CD209)	Tier 3B	Literature‐reported target
Q9NRJ3	C–C motif chemokine 28 (CCL28)	Tier 3B	/
Q9UHD0	Interleukin‐19 (IL19)	Tier 3A	Literature‐reported target
Q9UHY1	Nuclear receptor‐binding protein (NRBP1)	Tier 3B	/
Q9UIK4	Death‐associated protein kinase 2 (DAPK2)	Tier 1	/
Q9ULW2	Frizzled‐10 (FZD10)	Tier 3A	Clinical trial target

*Note:* Tier 1 includes efficacy targets of approved small molecules and biotherapeutic drugs as well as clinical‐phase drug candidates. Tier 2 encompasses genes encoding targets with known bioactive drug‐like small‐molecule binding partners as well as those with ≥ 50% identity (over 75% of the sequence) with approved drug targets. Tier 3 contains genes encoding secreted or extracellular proteins, proteins with more distant similarity to approved drug targets and members of key druggable gene families not already included in Tier 1 or 2. Within Tier 3, Tier 3A prioritizes genes that were in proximity (± 50 kbp) to a GWAS SNP and had an extracellular location. The remainder of the genes were assigned to Tier 3B.

Abbreviation: MRSC, a sequential stepwise analysis of Mendelian randomization, Steiger filtering test and colocalization.

^a^ Based on the druggable genes from Finan et al.

^b^ Based on the therapeutic target database.

In Table S29, we further annotated the drug target information for the 197 candidate biomarkers. Among these 197 proteins, 145 were identified by Finan et al. as druggable targets or supported by evidence from the TTD platform. Of these, 74 were both identified as druggable targets and supported by evidence from the TTD platform.

## 4 Discussion

To the best of our knowledge, this is the most comprehensive causal analysis of the relationship between the circulating proteome and sarcopenia‐related traits, with a rich array of multi‐layered analyses employed to enhance the reliability of the study results. In this study, according to the three key indicators (muscle mass, muscle strength and physical function) for diagnosing sarcopenia, we included nine sarcopenia‐related traits and 4372 circulating proteins (2508 unique proteins) with valid IVs. Our study applied a sequential stepwise MRSC screening method to precisely identify potential pathogenic proteins for sarcopenia. Through the MR analyses, we preliminarily identified a set of candidate causal proteins. Then, we conducted Steiger filtering tests and colocalization analyses to exclude reverse causality and eliminate the effect of LD between IVs and non‐IVs. Considering the possibility that filtering associations based on colocalization might lead to an underestimation of results [20], in order to minimize the risk of underestimation, we conducted two types of colocalization analyses and reported the final results based on the union of the two analyses. After these three steps of screening, 47 unique proteins were identified using only cis‐pQTLs and 79 unique proteins were identified using all the pQTLs.

Subsequently, in order to obtain more information about these identified causal proteins, we further conducted extensive analytical work, including annotation of PAVs, assessment of overlap between pQTLs and eQTLs, PPI analysis, pathway enrichment analysis and annotation of drug targets. It is worth noting that the causalities for proteins with PAVs as IVs should be interpreted with caution due to the presence of measurement artefacts and warrant further validation. The assessment of the overlap between pQTLs and eQTLs provided evidence on whether the association between SNP and protein was regulated by transcriptional level. Specifically, in our study, for proteins prioritized by MRSC analyses, the majority of cis‐pQTLs overlapped with their respective eQTLs and exhibited consistent directionality, whereas only one trans‐pQTL overlapped with an eQTL. This indicated that the cis‐pQTLs had greater biological interpretability for their causalities, whereas trans‐pQTLs possessed more complex regulatory mechanisms. PPI analyses indicated that the interactions among proteins prioritized by cis‐only MRSC and those prioritized by cis + trans MRSC were significantly higher than expected, suggesting potential relationships among these candidate proteins. Subsequently, pathway enrichment analyses further elucidated the enrichment of these interconnected proteins. The observed enrichment of these candidate proteins implied that they might interact in the pathogenesis of sarcopenia, thereby emphasizing the biological interpretability of our findings and the scientific validity of our candidate protein identification method. Furthermore, the observed enrichment also highlighted potential key pathways that could contribute to sarcopenia, such as the PI3K‐Akt signalling pathway, providing clues for future investigations into the mechanisms underlying sarcopenia and identifying effective drug targets. In this study, we annotated the identified causal proteins for their druggability, aiming to provide reference for the development of therapeutic drugs for sarcopenia or repurposing existing drugs for its treatment. It is noteworthy that among the 89 prioritized proteins in this study, 39 proteins utilized non‐PAVs as IVs and have supporting evidence as drug targets (Table S30). Given the substantial potential of these proteins as candidate drug targets for sarcopenia, their role in the pathogenesis of sarcopenia warrants further investigation.

Previously, two similar studies have been conducted to explore sarcopenia's causal proteins. Chen et al. [16] employed MR analysis to evaluate the associations between 310 plasma proteins and three sarcopenia‐related traits: appendicular lean mass (ALM), left hand grip strength and right hand grip strength. They ultimately identified five candidate causal proteins associated with sarcopenia, including LILRB2, ASPN, CNTN2, ART4 and SOD2. However, they did not distinguish between cis and trans for IVs, did not include an outcome of usual walking pace (a critical indicator of physical function) in the analyses and lacked in‐depth validation of the reliability of causal associations, such as the colocalization analysis. Jiang et al. [39] performed only cis‐MR analyses, including 72 circulating proteins as exposures, and ultimately identified five candidate causal proteins (HP, ATP1B2, ISLR2, TNFSF12 and HGF) associated with sarcopenia. Similarly, they did not consider the usual walking pace, did not assess reverse causality and lacked in‐depth discussion of the reliability of causal associations. In our study, we also observed evidence of HP, ART4 and TNFSF12 as candidate pathogenic proteins for sarcopenia, and the direction of the associations obtained is consistent with the results reported in the aforementioned two studies.

The measurement of the circulating proteome by the UKB afforded an opportunity to explore disease biomarkers. In this study, 45 proteins provided by the UKB overlapped with those prioritized by MRSC analyses, and among these, only two proteins (CTSB and ASGR1) were found to be significant in the linear regression analyses. The observed discrepancy could be attributed to the influence of confounding factors on observational studies, which often hinder the identification of objective associations. Moreover, since the observational study cannot establish causality, we cannot definitively conclude that the 197 proteins identified through linear regression are causal proteins for sarcopenia. Instead, they could only be considered as putative biomarkers for sarcopenia. Nonetheless, the integration of MR analyses with the results from observational studies could provide more clues for the in‐depth exploration of candidate pathogenic proteins and drug targets for sarcopenia.

CTSB is a myokine that has previously been shown to be upregulated in the muscle of patients with Duchenne muscular dystrophy, mainly localized to areas of macrophage infiltration [40]. When monitoring muscle pathology in a mouse model of myopathy, Baudy et al. found that CTSB imaging showed localization in inflammatory infiltrates and regenerating muscle fibres, and its activity and mRNA levels peaked at the junction of inflammation and myoblast fusion [41]. Our MR analysis observed that CTSB was negatively associated with the whole body FFM, suggesting that it is a potential risk factor for sarcopenia. Besides, the observational study derived from the UKB data has also enhanced the strength of evidence supporting this conclusion. As a preclinical target for hair loss and bone cancer, the potential of CTSB as a drug target for sarcopenia warrants further validation.

It has been reported that the deficiency of ASGR1 may alleviate diet‐induced systemic insulin resistance via improved hepatic insulin sensitivity [42]. Given the close relationship between insulin resistance and sarcopenia, it is hypothesized that this protein may affect the metabolism of muscle tissue. Our MR analysis found that ASGR1 was positively correlated with the sarcopenia‐related traits. Based on the UKB data, the observational study has provided additional evidence supporting this conclusion. Currently, a novel anti‐ASGR1 monoclonal antibody named AMG 529 has been developed for the treatment of cardiovascular diseases, but it remains in the clinical research stage. In the future, it would be meaningful to further explore the association between this protein and sarcopenia.

For the 89 candidate pathogenic proteins prioritized by MRSC, a comprehensive literature review was conducted to examine the evidence of their associations with sarcopenia/myopathy/skeletal muscle physiology, including evidence from population‐based association analyses, MR analyses and mechanism research. We observed that 38 proteins (Table S31) had been previously reported to be associated with sarcopenia. Among these, 20 proteins exhibited clear evidence that supported the same direction of association as determined by the MR analyses, thereby suggesting a notable potential for these 20 proteins as therapeutic targets for sarcopenia. For the remaining 18 proteins, the majority lacked adequate previous evidence to support the direction of their associations, with only a few proteins having their associations still in dispute, warranting further investigation.

The limitations of this study are summarized as follows: First, the circulating proteins analysed in this study encompass those that are purposefully secreted as well as those that have leaked. The abundance of these circulating proteins may vary from that of proteins within cells and tissues. Thus, the potential impact of cell‐ or tissue‐specific protein abundance has not been explored. Second, the proteins identified with PAVs as IVs might lead to wrong conclusions due to the existence of measurement artefacts. However, the PAV assessment conducted on cis‐pQTLs offered clues to this problem. Last, the causal associations identified in this study have not been validated through animal experiments or other mechanism explorations.

In conclusion, our study identified 89 potential pathogenic proteins and 197 candidate biomarkers for sarcopenia, including both previously reported and newly discovered. More than half of these proteins are drug targets or druggable. Our findings contribute to a deeper understanding of the pathophysiology of sarcopenia, providing valuable clues for the development of specific therapeutic drugs for sarcopenia.

## Conflicts of Interest

The authors declare no conflicts of interest.

## Supporting information


**Figure S1** Protein–protein interaction (PPI) networks of proteins.
**Figure S2.** Bar plot of the Reactome pathway enrichment analysis.
**Figure S3.** Bar plot of the KEGG pathway enrichment analysis.
**Figure S4.** Bar plot of the GO pathway enrichment analysis.

**Table S1** Information of the exposures.
Table S2.Information of the outcomes.
Table S3.Bonferroni *P*‐value threshold for each outcome.
Table S4.Information of the instrumental variables.
Table S5.Main analyses results of the cis‐only MR.
Table S6. Associations of cis‐only MR‐prioritized proteins with the outcomes.
Table S7.Sensitivity analyses results of cis‐only MR.
Table S8.Significant results of the sensitivity analyses of cis‐only MR.
Table S9.Main analyses results of the cis+trans MR.
Table S10.Associations of cis+trans MR‐prioritized proteins with the outcomes.
Table S11.Sensitivity analyses results of cis+trans MR.
Table S12.Significant results of the sensitivity analyses of cis+trans MR.
Table S13.Steiger filtering analysis for associations identified by main analyses of cis‐only MR.
Table S14.Steiger filtering analysis for associations identified by main analyses of cis+trans MR.
Table S15.Colocalization of the pQTLs prioritized by cis‐only MR and Steiger filtering analysis.
Table S16.Colocalization of the pQTLs prioritized by cis+trans MR and Steiger filtering analysis.
Table S17.PAV assessment for the cis‐pQTLs prioritized by cis‐only MRSC analysis.
Table S18.Overlaping of cis‐only MR‐prioritized pQTLs with eQTLs.
Table S19.Overlaping of cis+trans MR‐prioritized pQTLs with eQTLs.
Table S20.Significantly enriched pathways of Reactome pathway enrichment analysis for proteins prioritized by cis‐only MRSC.
Table S21.Significantly enriched pathways of KEGG pathway enrichment analysis for proteins prioritized by cis‐only MRSC.
Table S22.Significantly enriched pathways of GO pathway enrichment analysis for proteins prioritized by cis‐only MRSC.
Table S23.Significantly enriched pathways of GO pathway enrichment analysis for proteins prioritized by cis+trans MRSC.
Table S24.Linear regression analyses examining the association between protein levels and the longitudinal change levels of sarcopenia‐related traits (Data provided by UK Biobank).
Table S25.Candidate biomarkers of sarcopenia identified by linear regression analysis.
Table S26.The overlap between proteins from the UK Biobank and proteins prioritized by the cis‐only MRSC.
Table S27.The overlap between proteins from the UK Biobank and proteins prioritized by the cis+trans MRSC.
Table S28.Drug target annotations of the proteins prioritized by MRSC.
Table S29.Drug target annotations of the proteins as candidate biomarker for sarcopenia.
Table S30.Proteins prioritized by MRSC using non‐PAVs as IVs and have supporting evidence for drug targets.
Table S31.Evidence from previous studies on proteins prioritized by MRSC.

## Data Availability

Summary data involved in the MR analysis can be accessed and downloaded from original studies. Researchers registered with the UK Biobank can apply for access to its resources by visiting https://www.ukbiobank.ac.uk/enable‐your‐research/register.

## References

[1] E. Marzetti , R. Calvani , M. Tosato , et al., “Sarcopenia: An Overview,” Aging Clinical and Experimental Research 29, no. 1 (2017): 11–17, 10.1007/s40520-016-0704-5. 28155183

[2] A. J. Mayhew , K. Amog , S. Phillips , et al., “The Prevalence of Sarcopenia in Community‐Dwelling Older Adults, an Exploration of Differences Between Studies and Within Definitions: A Systematic Review and meta‐Analyses,” Age and Ageing 48, no. 1 (2019): 48–56, 10.1093/ageing/afy106. 30052707

[3] A. J. Cruz‐Jentoft , J. P. Baeyens , J. M. Bauer , et al., “Sarcopenia: European Consensus on Definition and Diagnosis: Report of the European Working Group on Sarcopenia in Older People,” Age and Ageing 39, no. 4 (2010): 412–423, 10.1093/ageing/afq034. 20392703 PMC2886201

[4] L. K. Chen , J. Woo , P. Assantachai , et al., “Asian Working Group for Sarcopenia: 2019 Consensus Update on Sarcopenia Diagnosis and Treatment,” Journal of the American Medical Directors Association 21, no. 3 (2020): 300–307.e302, 10.1016/j.jamda.2019.12.012. 32033882

[5] A. A. Sayer , H. Syddall , H. Martin , H. Patel , D. Baylis , and C. Cooper , “The Developmental Origins of Sarcopenia,” Journal of Nutrition, Health & Aging 12, no. 7 (2008): 427–432, 10.1007/bf02982703. PMC2652119 18615224

[6] A. A. Sayer , C. Stewart , H. Patel , and C. Cooper , “The Developmental Origins of Sarcopenia: From Epidemiological Evidence to Underlying Mechanisms,” Journal of Developmental Origins of Health and Disease 1, no. 3 (2010): 150–157, 10.1017/s2040174410000097. 25141783

[7] T. Wu , X. Yan , Y. Liu , et al., “Association Between Early Life Exposure to the Great Famine and Possible Sarcopenia in Older Chinese Adults: A National Cross‐Sectional Study,” BMJ Open 13, no. 3 (2023): e065240, 10.1136/bmjopen-2022-065240. PMC9980362 36858468

[8] H. Jin , H. J. Yoo , Y. A. Kim , et al., “Unveiling Genetic Variants for Age‐Related Sarcopenia by Conducting a Genome‐Wide Association Study on Korean Cohorts,” Scientific Reports 12, no. 1 (2022): 3501, 10.1038/s41598-022-07567-9. 35241739 PMC8894365

[9] S. E. Wu and W. L. Chen , “A Genome‐Wide Association Study Identifies Novel Risk Loci for Sarcopenia in a Taiwanese Population,” Journal of Inflammation Research 14 (2021): 5969–5980, 10.2147/jir.S338724. 34815687 PMC8605878

[10] G. Jones , K. Trajanoska , A. J. Santanasto , et al., “Genome‐Wide Meta‐Analysis of Muscle Weakness Identifies 15 Susceptibility Loci in Older Men and Women,” Nature Communications 12, no. 1 (2021): 654, 10.1038/s41467-021-20918-w. PMC7844411 33510174

[11] S. M. Willems , D. J. Wright , F. R. Day , et al., “Large‐Scale GWAS Identifies Multiple Loci for Hand Grip Strength Providing Biological Insights Into Muscular Fitness,” Nature Communications 8 (2017): 16015, 10.1038/ncomms16015. PMC5510175 29313844

[12] C. Corbo , A. A. Li , H. Poustchi , et al., “Analysis of the Human Plasma Proteome Using Multi‐Nanoparticle Protein Corona for Detection of Alzheimer's Disease,” Advanced Healthcare Materials 10, no. 2 (2021): e2000948, 10.1002/adhm.202000948. 33169521

[13] P. E. Geyer , N. A. Kulak , G. Pichler , L. M. Holdt , D. Teupser , and M. Mann , “Plasma Proteome Profiling to Assess Human Health and Disease,” Cell Systems 2, no. 3 (2016): 185–195, 10.1016/j.cels.2016.02.015. 27135364

[14] Y. Chen , S. Liu , W. Gong , et al., “Protein‐Centric Omics Integration Analysis Identifies Candidate Plasma Proteins for Multiple Autoimmune Diseases,” Human Genetics 143 (2023): 1035–1048, 10.1007/s00439-023-02627-0. 38143258 PMC11485194

[15] T. Jin , M. Wang , Z. Zeng , et al., “Causal Associations of Plasma Omega‐3 Polyunsaturated Fatty Acids With Sarcopenia‐Related Traits: A Two‐Sample Mendelian Randomization Study,” European Journal of Clinical Nutrition 78 (2023): 19–26, 10.1038/s41430-023-01339-y. 37653236

[16] B. B. Chen , J. Q. Wang , X. H. Meng , et al., “Putative Candidate Drug Targets for Sarcopenia‐Related Traits Identified Through Mendelian Randomization Analysis of the Blood Proteome,” Frontiers in Genetics 13 (2022): 923429, 10.3389/fgene.2022.923429. 35938019 PMC9354522

[17] S. Schluessel , W. Zhang , H. Nowotny , et al., “11‐beta‐Hydroxysteroid Dehydrogenase Type 1 (HSD11B1) Gene Expression in Muscle Is Linked to Reduced Skeletal Muscle Index in Sarcopenic Patients,” Aging Clinical and Experimental Research 35, no. 12 (2023): 3073–3083, 10.1007/s40520-023-02574-w. 37943405 PMC10721692

[18] H. Cui , D. Hu , Y. Liu , and J. Zhao , “Identifying Acss1, Mtfp1 and Oxct1 as Key Regulators and Promising Biomarkers of Sarcopenia in Various Models,” Gene 896 (2024): 148053, 10.1016/j.gene.2023.148053. 38042218

[19] S. J. Park , E. Ji , H. J. Yoo , et al., “Circulating Lumican as a Potential Biomarker for Osteosarcopenia in Older Adults,” Bone 179 (2024): 116959, 10.1016/j.bone.2023.116959. 37956822

[20] J. Zheng , V. Haberland , D. Baird , et al., “Phenome‐Wide Mendelian Randomization Mapping the Influence of the Plasma Proteome on Complex Diseases,” Nature Genetics 52, no. 10 (2020): 1122–1131, 10.1038/s41588-020-0682-6. 32895551 PMC7610464

[21] Y. Zhang , J. Xie , S. Wen , et al., “Evaluating the Causal Effect of Circulating Proteome on the Risk of Osteoarthritis‐Related Traits,” Annals of the Rheumatic Diseases 82, no. 12 (2023): 1606–1617, 10.1136/ard-2023-224459. 37595989

[22] S. C. Larsson , A. S. Butterworth , and S. Burgess , “Mendelian Randomization for Cardiovascular Diseases: Principles and Applications,” European Heart Journal 44, no. 47 (2023): 4913–4924, 10.1093/eurheartj/ehad736. 37935836 PMC10719501

[23] K. Suhre , M. Arnold , A. M. Bhagwat , et al., “Connecting Genetic Risk to Disease End Points Through the Human Blood Plasma Proteome,” Nature Communications 8 (2017): 14357, 10.1038/ncomms14357. PMC5333359 28240269

[24] L. Folkersen , E. Fauman , M. Sabater‐Lleal , et al., “Mapping of 79 Loci for 83 Plasma Protein Biomarkers in Cardiovascular Disease,” PLoS Genetics 13, no. 4 (2017): e1006706, 10.1371/journal.pgen.1006706. 28369058 PMC5393901

[25] B. B. Sun , J. C. Maranville , J. E. Peters , et al., “Genomic Atlas of the Human Plasma Proteome,” Nature 558, no. 7708 (2018): 73–79, 10.1038/s41586-018-0175-2. 29875488 PMC6697541

[26] R. F. Hillary , D. L. McCartney , S. E. Harris , et al., “Genome and Epigenome Wide Studies of Neurological Protein Biomarkers in the Lothian Birth Cohort 1936,” Nature Communications 10, no. 1 (2019): 3160, 10.1038/s41467-019-11177-x. PMC6639385 31320639

[27] M. Pietzner , E. Wheeler , J. Carrasco‐Zanini , et al., “Genetic Architecture of Host Proteins Involved in SARS‐CoV‐2 Infection,” Nature Communications 11, no. 1 (2020): 6397, 10.1038/s41467-020-19996-z. PMC7744536 33328453

[28] A. Gilly , Y. C. Park , G. Png , et al., “Whole‐Genome Sequencing Analysis of the Cardiometabolic Proteome,” Nature Communications 11, no. 1 (2020): 6336, 10.1038/s41467-020-20079-2. PMC7729872 33303764

[29] E. Ferkingstad , P. Sulem , B. A. Atlason , et al., “Large‐Scale Integration of the Plasma Proteome With Genetics and Disease,” Nature Genetics 53, no. 12 (2021): 1712–1721, 10.1038/s41588-021-00978-w. 34857953

[30] A. Gudjonsson , V. Gudmundsdottir , G. T. Axelsson , et al., “A Genome‐Wide Association Study of Serum Proteins Reveals Shared Loci With Common Diseases,” Nature Communications 13, no. 1 (2022): 480, 10.1038/s41467-021-27850-z. PMC8789779 35078996

[31] C. Sudlow , J. Gallacher , N. Allen , et al., “UK Biobank: An Open Access Resource for Identifying the Causes of a Wide Range of Complex Diseases of Middle and Old Age,” PLoS Medicine 12, no. 3 (2015): e1001779, 10.1371/journal.pmed.1001779. 25826379 PMC4380465

[32] S. Burgess , A. Butterworth , and S. G. Thompson , “Mendelian Randomization Analysis With Multiple Genetic Variants Using Summarized Data,” Genetic Epidemiology 37, no. 7 (2013): 658–665, 10.1002/gepi.21758. 24114802 PMC4377079

[33] J. Bowden , G. Davey Smith , and S. Burgess , “Mendelian Randomization With Invalid Instruments: Effect Estimation and bias Detection Through Egger Regression,” International Journal of Epidemiology 44, no. 2 (2015): 512–525, 10.1093/ije/dyv080. 26050253 PMC4469799

[34] S. Burgess and S. G. Thompson , “Interpreting Findings From Mendelian Randomization Using the MR‐Egger Method,” European Journal of Epidemiology 32, no. 5 (2017): 377–389, 10.1007/s10654-017-0255-x. 28527048 PMC5506233

[35] J. Bowden , G. Davey Smith , P. C. Haycock , and S. Burgess , “Consistent Estimation in Mendelian Randomization With Some Invalid Instruments Using a Weighted Median Estimator,” Genetic Epidemiology 40, no. 4 (2016): 304–314, 10.1002/gepi.21965. 27061298 PMC4849733

[36] C. Wallace , “A More Accurate Method for Colocalisation Analysis Allowing for Multiple Causal Variants,” PLoS Genetics 17, no. 9 (2021): e1009440, 10.1371/journal.pgen.1009440. 34587156 PMC8504726

[37] Y. Zhou , Y. Zhang , D. Zhao , et al., “TTD: Therapeutic Target Database Describing Target Druggability Information,” Nucleic Acids Research 52, no. D1 (2024): D1465–d1477, 10.1093/nar/gkad751. 37713619 PMC10767903

[38] C. Finan , A. Gaulton , F. A. Kruger , et al., “The Druggable Genome and Support for Target Identification and Validation in Drug Development,” Science Translational Medicine 9, no. 383 (2017): eaag1166, 10.1126/scitranslmed.aag1166. 28356508 PMC6321762

[39] W. Jiang , W. Zhan , L. Zhou , et al., “Potential Therapeutic Targets for Sarcopenia Identified by Mendelian Randomisation,” Age and Ageing 52, no. 2 (2023): afad024, 10.1093/ageing/afad024. 36821647 PMC9949583

[40] D. T. Jane , L. Morvay , L. Dasilva , D. Cavallo‐Medved , B. F. Sloane , and M. J. Dufresne , “Cathepsin B Localizes to Plasma Membrane Caveolae of Differentiating Myoblasts and Is Secreted in an Active Form at Physiological pH,” Biological Chemistry 387, no. 2 (2006): 223–234, 10.1515/bc.2006.030. 16497156

[41] A. R. Baudy , A. Sali , S. Jordan , et al., “Non‐Invasive Optical Imaging of Muscle Pathology in mdx Mice Using Cathepsin Caged Near‐Infrared Imaging,” Molecular Imaging and Biology 13, no. 3 (2011): 462–470, 10.1007/s11307-010-0376-z. 20661652 PMC3087873

[42] X. Yu , J. Tao , Y. Wu , et al., “Deficiency of ASGR1 Alleviates Diet‐Induced Systemic Insulin Resistance via Improved Hepatic Insulin Sensitivity,” Diabetes and Metabolism Journal 48 (2024): 802–815, 10.4093/dmj.2023.0124. 38310881 PMC11307118

