# Supplementary material for: Association Analysis of the Circulating Proteome With Sarcopenia‐Related Traits Reveals Potential Drug Targets for Sarcopenia
Source: J Cachexia Sarcopenia Muscle. 2025 Feb 13;16(1):e13720. doi: 10.1002/jcsm.13720 (PMC11825984; doi:10.1002/jcsm.13720)
Supplement: Supplementary file 1 — Figure S1 Protein–protein interaction (PPI) networks of proteins. Figure S2. Bar plot of the Reactome pathway enrichment analysis. Figure S3. Bar plot of the KEGG pathway enrichment analysis. Figure S4. Bar plot of the GO pathway enrichment analysis. [file JCSM-16-e13720-s001.docx]

**CONTENTS**

| Figure S1. Protein–protein interaction (PPI) networks of proteins | 2 |
| --- | --- |
| Figure S2. Bar plot of the Reactome pathway enrichment analysis | 4 |
| Figure S3. Bar plot of the KEGG pathway enrichment analysis | 5 |
| Figure S4. Bar plot of the GO pathway enrichment analysis | 6 |

**Supplementary Figure S1. Protein–protein interaction (PPI) networks of proteins**


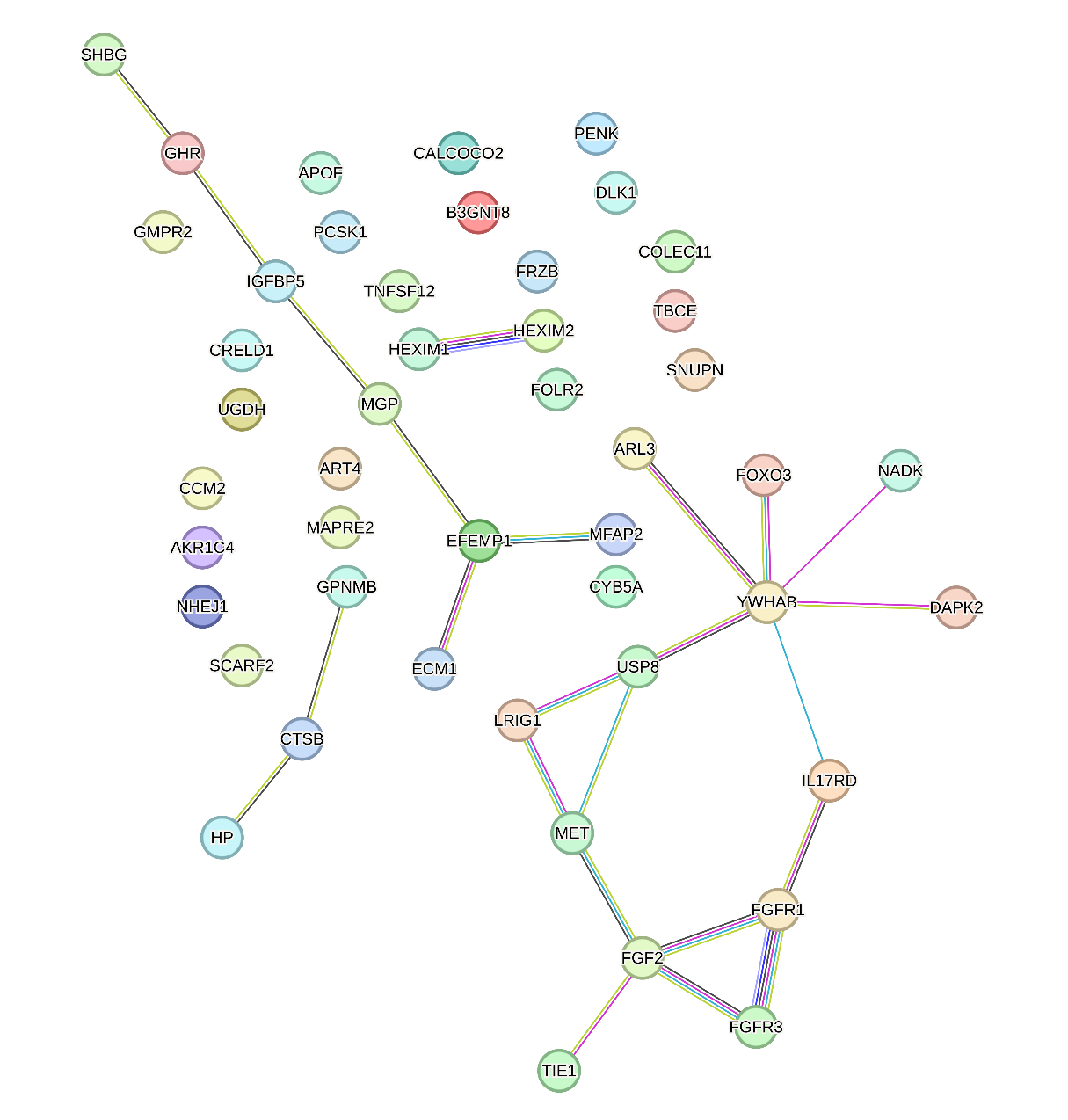
**A**


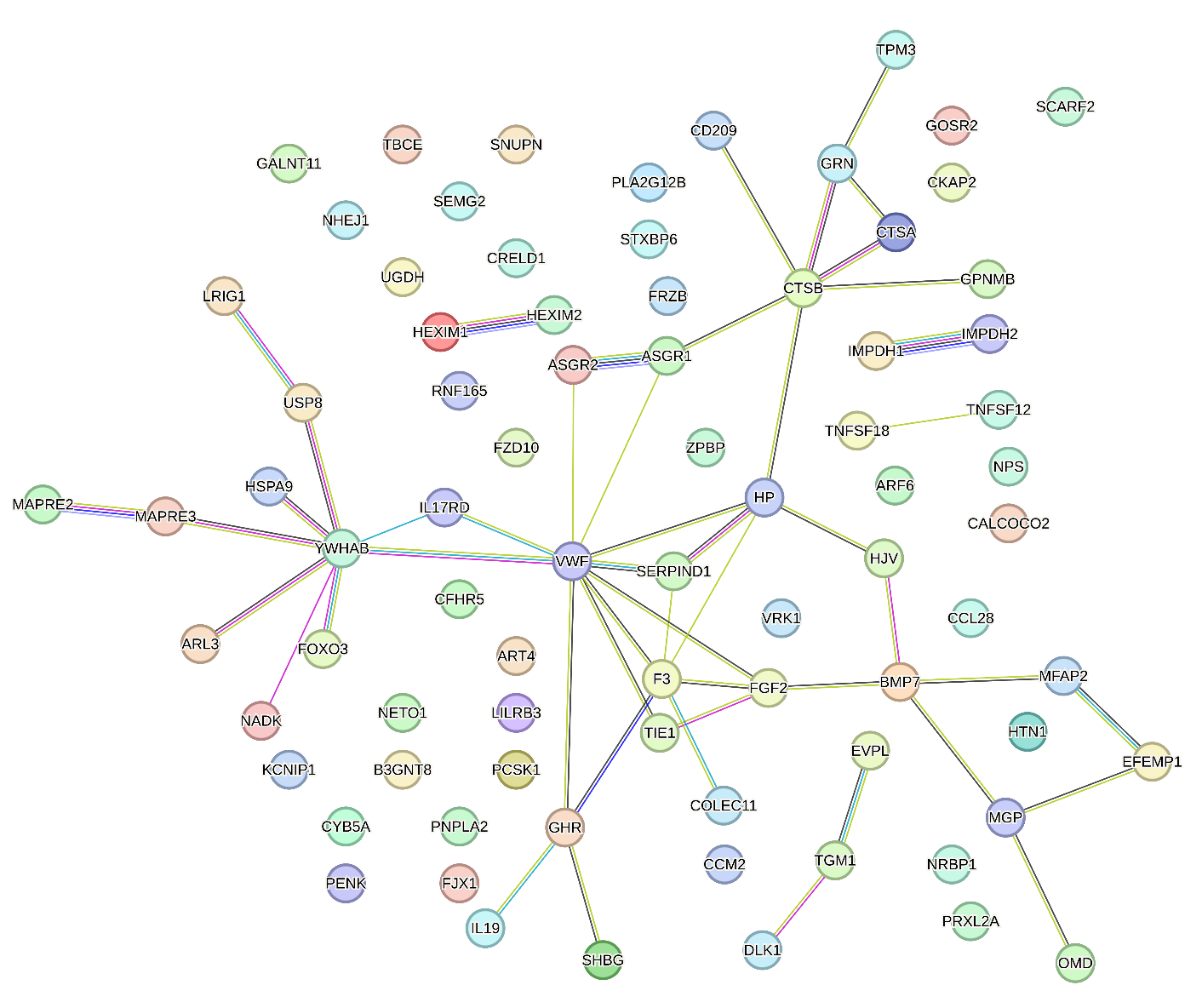
**B**


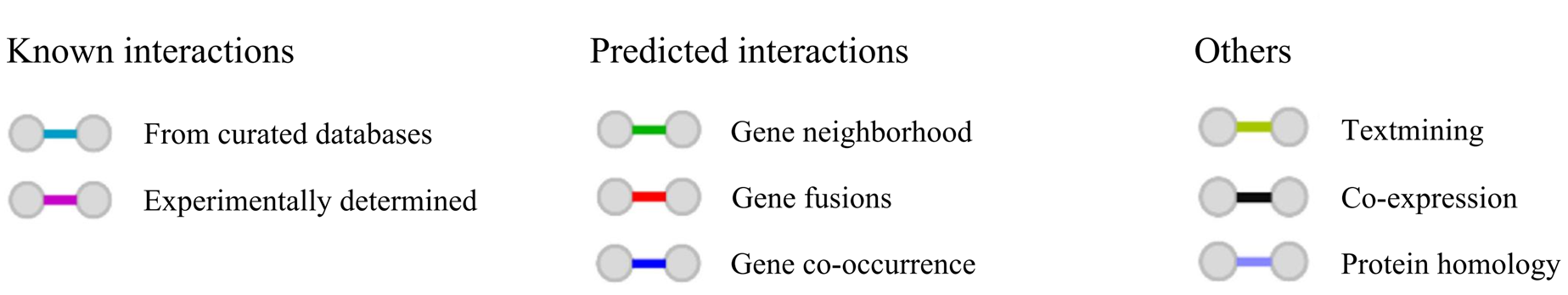


(A) PPI network of the proteins prioritized by cis-only MRSC. By using the interaction score threshold of 0.4 (medium confidence), the STRING PPI analysis yielded a clustered network with clustering coefficient of 0.323. The PPI network contained 47 nodes with 24 edges (expected number of edges: 11), indicating this network has significantly more interactions than expected (enrichment *P* value=0.000404). (B) PPI network of the proteins prioritized by cis+trans MRSC. By using the interaction score threshold of 0.4 (medium confidence), the STRING PPI analysis yielded a clustered network with clustering coefficient of 0.355. The PPI network contained 79 nodes with 50 edges (expected number of edges: 28), indicating this network has significantly more interactions than expected (enrichment *P* value=8.76×10^-5^).

**Supplementary Figure S2. Bar plot of the Reactome pathway enrichment analysis**

**A**


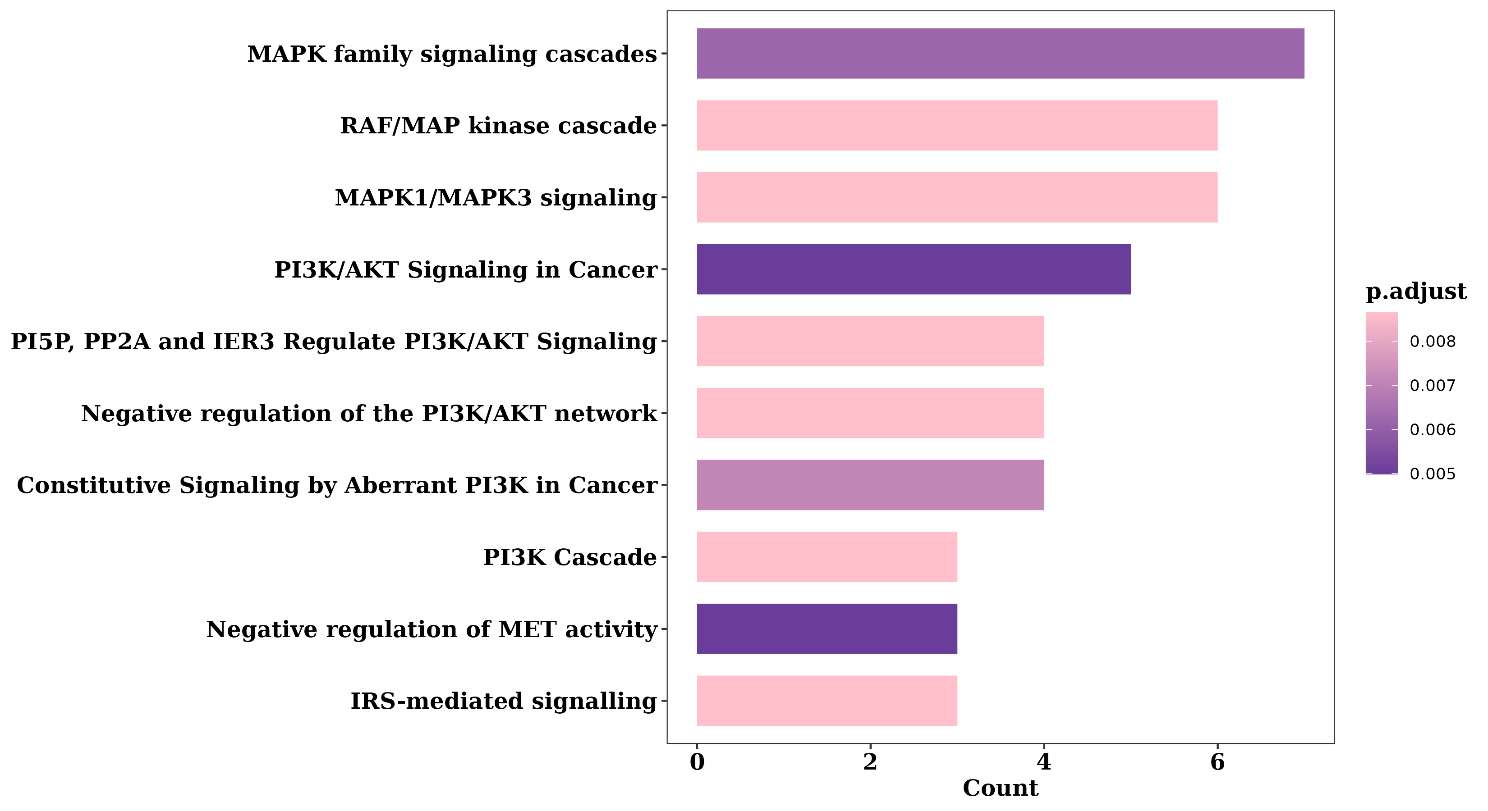


**B**


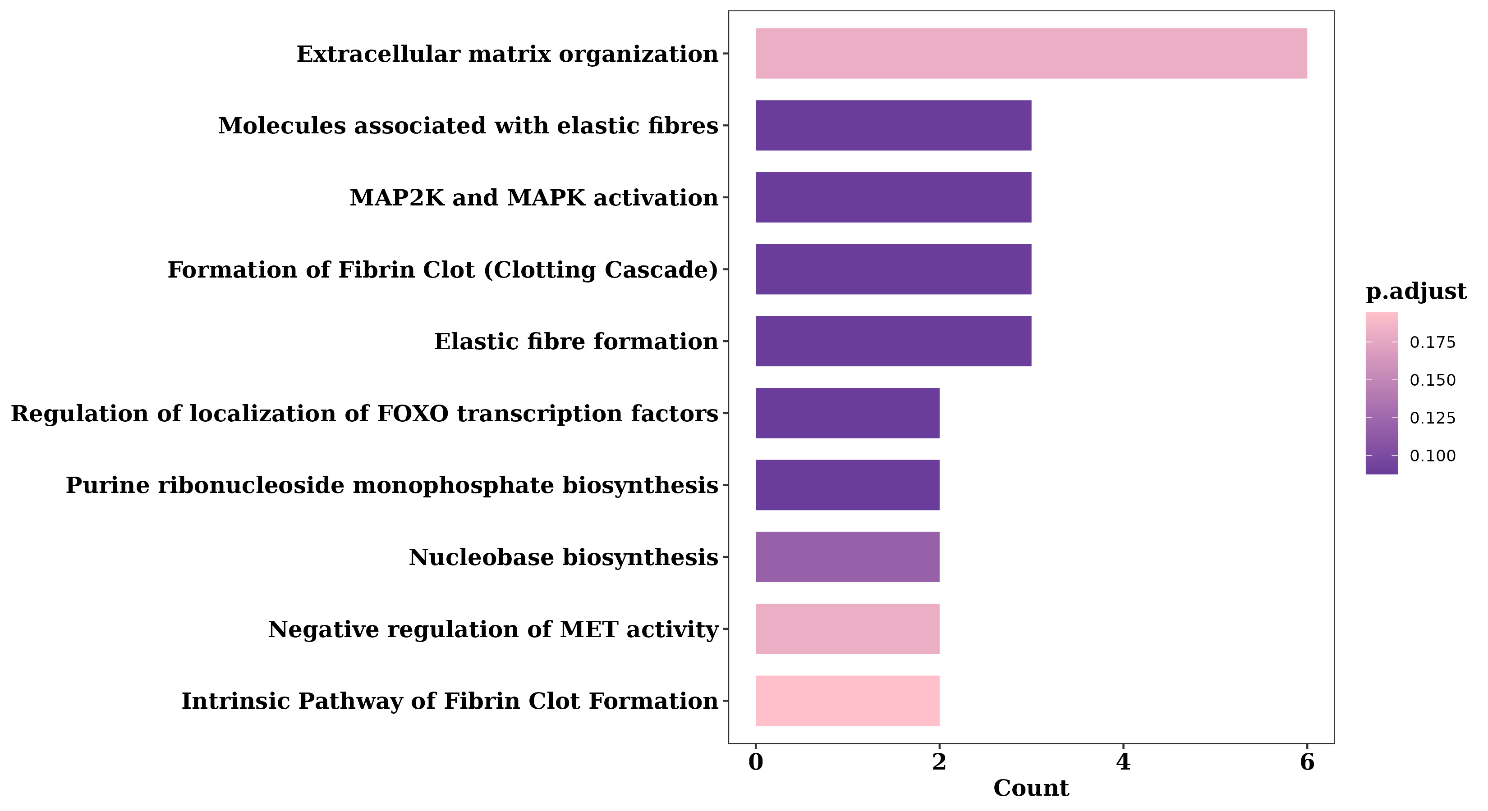
(A) The top 10 most significant enriched pathways for proteins prioritized by cis-only MRSC. (B) The top 10 most significant enriched pathways for proteins prioritized by cis+trans MRSC.

**Supplementary Figure S3. Bar plot of the KEGG pathway enrichment analysis**

**A**


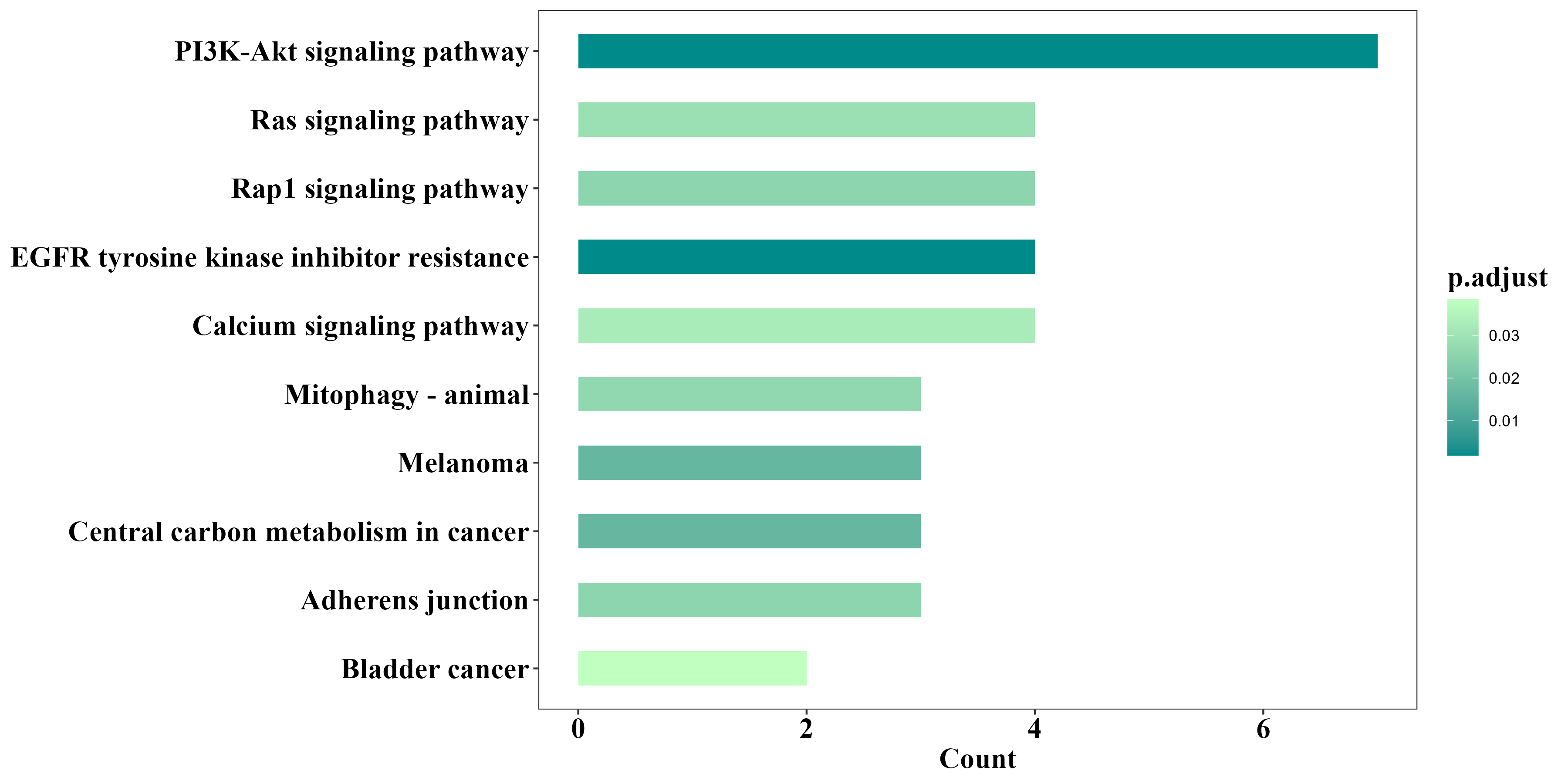


**B**


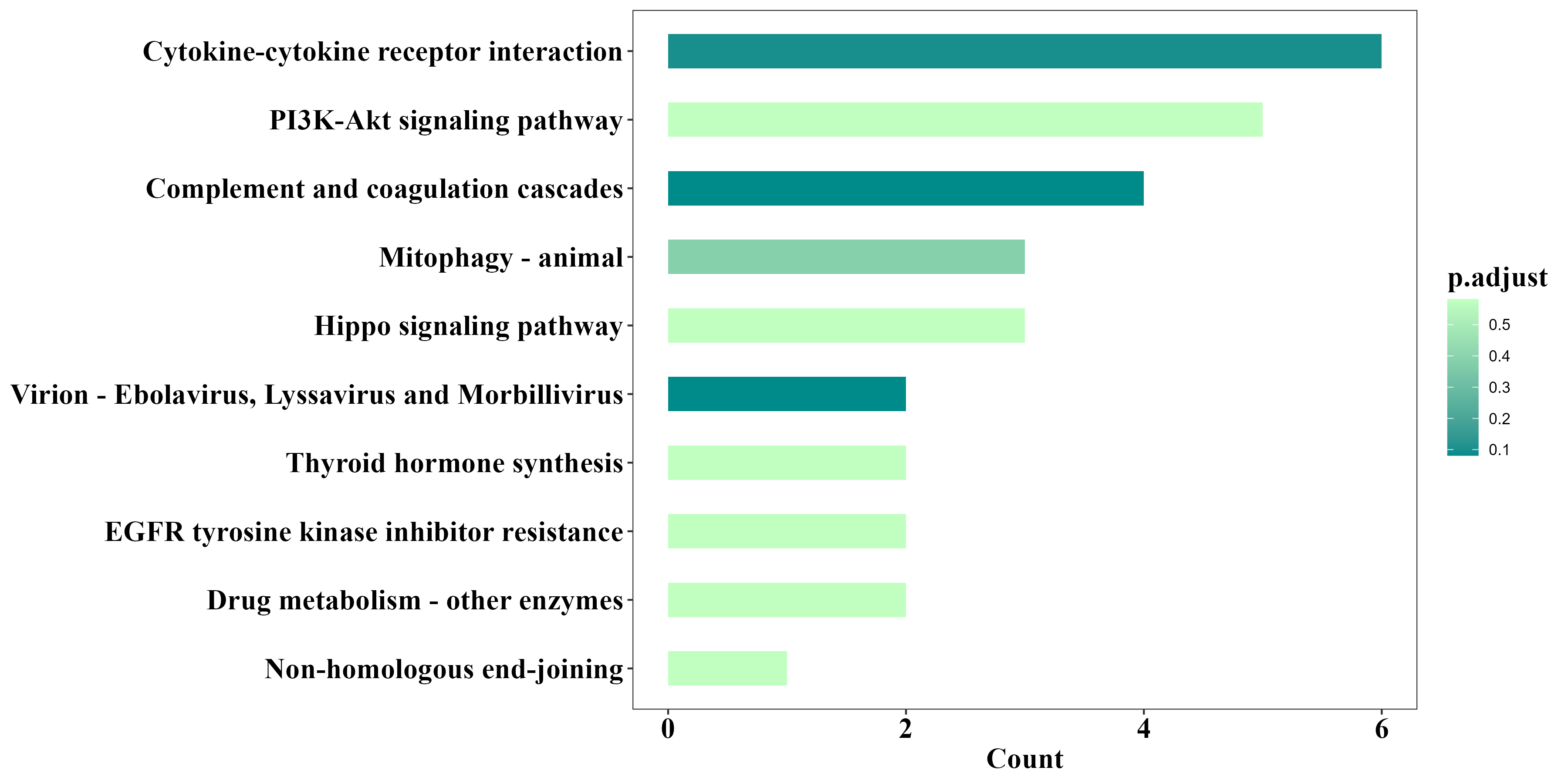
(A) The top 10 most significant enriched pathways for proteins prioritized by cis-only MRSC. (B) The top 10 most significant enriched pathways for proteins prioritized by cis+trans MRSC.

**Supplementary Figure S4. Bar plot of the GO pathway enrichment analysis**

**A**


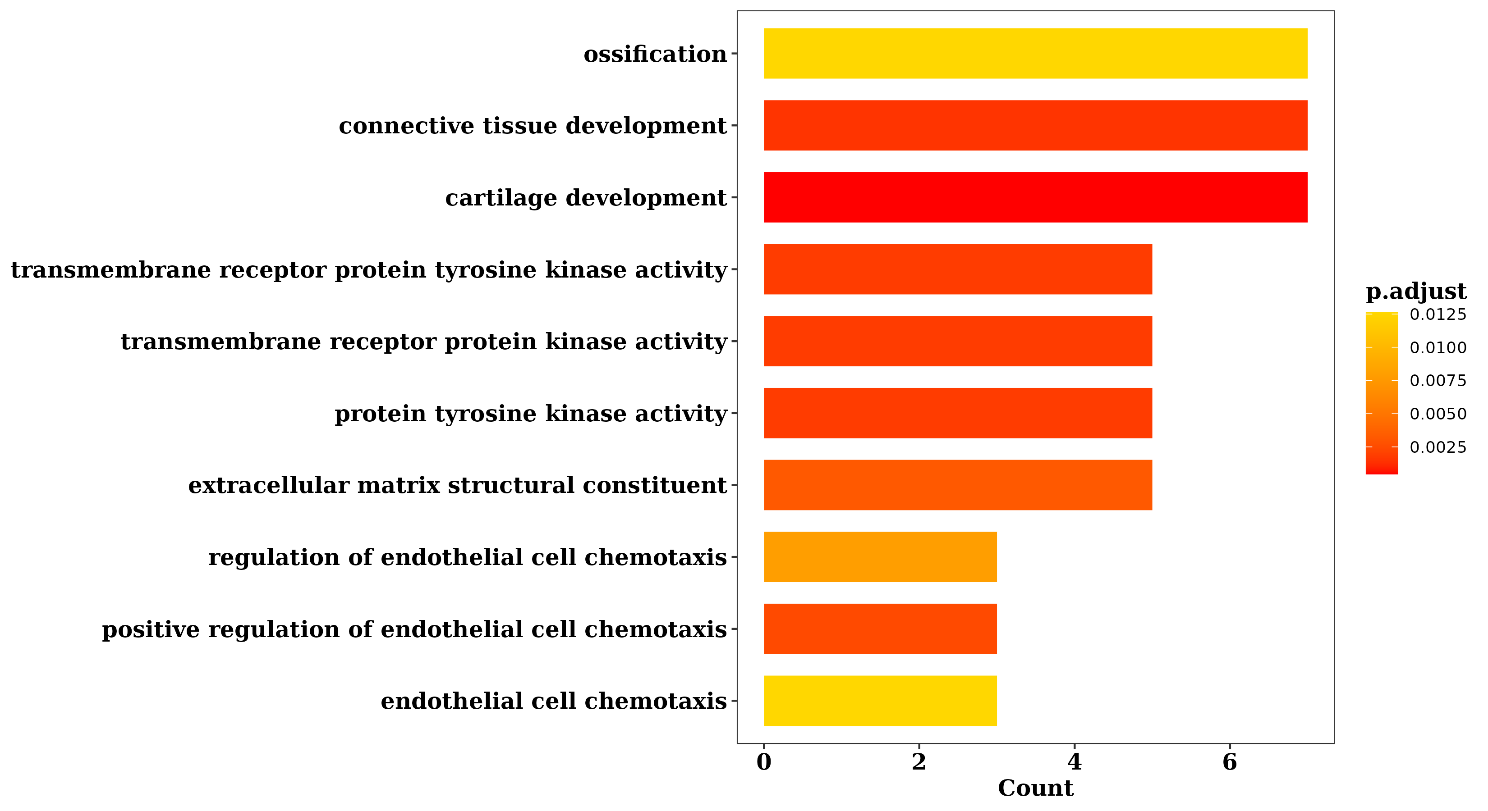


**B**


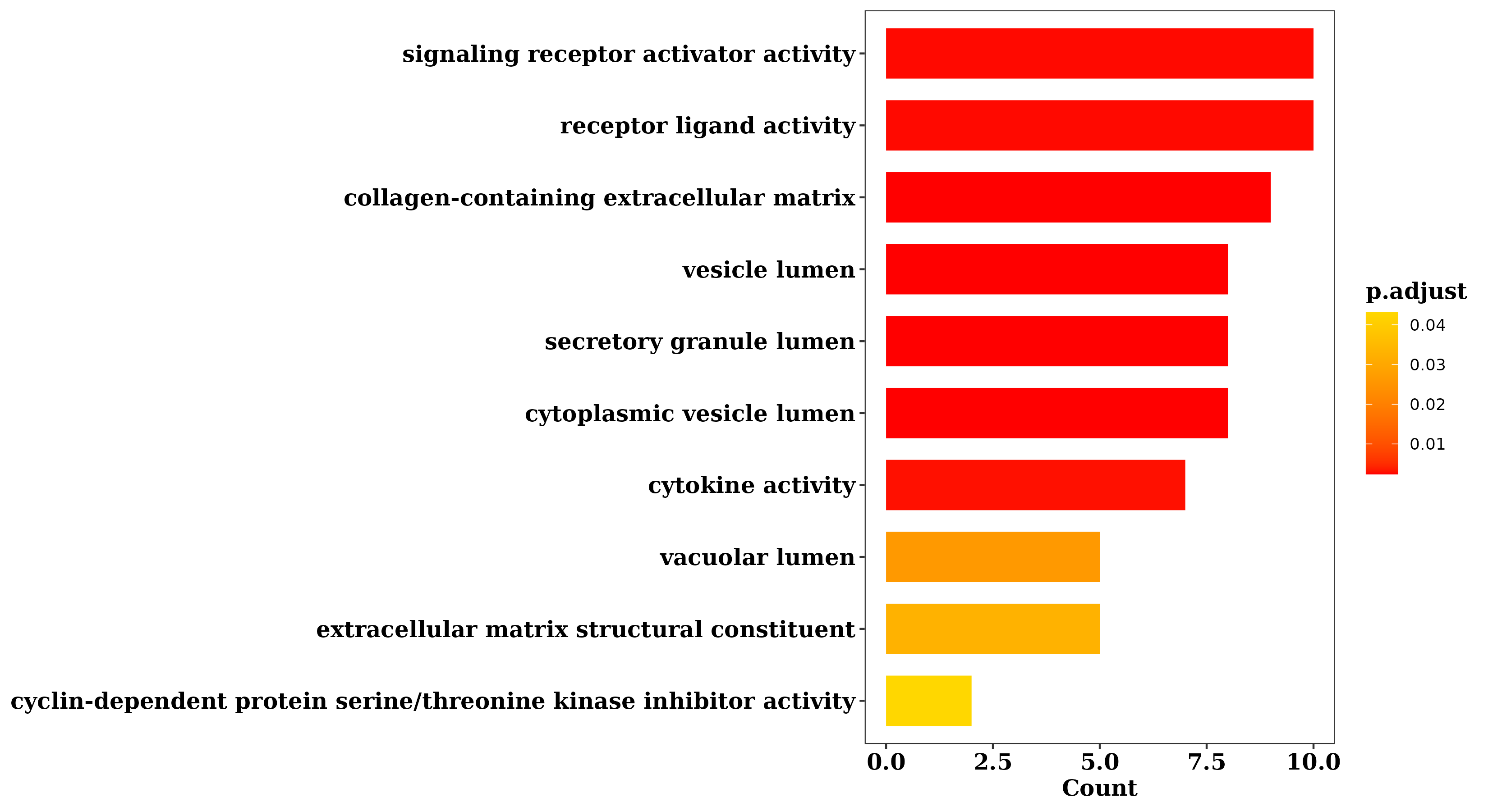
(A) The top 10 most significant enriched pathways for proteins prioritized by cis-only MRSC. (B) The top 10 most significant enriched pathways for proteins prioritized by cis+trans MRSC.
